# Taxonomic notes on eleven species of the subfamily Cteninae (Araneae, Ctenidae) from Asia

**DOI:** 10.3897/BDJ.10.e96003

**Published:** 2022-12-12

**Authors:** Chang Chu, Ying Lu, Shuqiang Li, Zhiyuan Yao

**Affiliations:** 1 College of Life Science, Shenyang Normal University, Shenyang, China College of Life Science, Shenyang Normal University Shenyang China; 2 Institute of Zoology, Chinese Academy of Sciences, Beijing, China Institute of Zoology, Chinese Academy of Sciences Beijing China

**Keywords:** biodiversity, DNA barcode, morphology, new species, tropics and subtropics

## Abstract

**Background:**

The spider family Ctenidae Keyserling, 1877 has a worldwide distribution with 584 species belonging to 49 genera. Amongst these, 141 species are from Asia, including 130 species assigned to Cteninae Keyserling, 1877.

**New information:**

Nine new species belonging to three genera of Cteninae are reported from Asia: *Amauropelmakrabi* sp. n. (female; Krabi, Thailand), *Am.phangnga* sp. n. (male; Phang Nga, Thailand), *Am.saraburi* sp. n. (male and female; Saraburi, Thailand); *Anahitamedog* sp. n. (male and female; Tibet, China); *Bowieninhbinh* sp. n. (male; Ninh Binh, Vietnam) and *B.vinhphuc* sp. n. (male and female; Vinh Phuc, Vietnam) from the *robustus*-species group; *B.borneo* sp. n. (male; Sabah, Malaysia) from the *chinagirl*-species group; *B.engkilili* sp. n. (female; Engkilili, Malaysia); *B.sabah* sp. n. (male and female; Sabah, Malaysia) from the *scarymonsters*-species group. The male of *An.popa* Jäger & Minn, 2015 and the female of *B.fascination* Jäger, 2022 (*robustus*-species group) are described for the first time. *B.fascination* Jäger, 2022 is reported from China for the first time. In addition, the DNA barcodes of all the species in this study were obtained, except for *B.vinhphuc* sp. n.

## Introduction

The spider family Ctenidae Keyserling, 1877 has a worldwide distribution, but mainly occurs in the tropical and subtropical regions (Jäger 2022). Instead of building webs to catch prey, they are free-hunting nocturnal spiders and hide in litter layers, small cracks in soil, tree trunks and low vegetation during the day (Polotow and Jocqué 2014, Jäger 2022), whilst a few species are cave-dwelling (Jäger 2012, Miller and Rahmadi 2012, Lin et al. 2022). Therefore, they are often called wandering spiders (Jocqué and Dippenaar-Schoeman 2006, Polotow and Brescovit 2018, Jäger 2022). Ctenidae currently contains 49 genera and 584 species, accounting for about one percent of the known diversity of all spiders (Jäger 2022, World Spider Catalog 2022). It is composed of four subfamilies: Acantheinae Simon, 1897, Acanthocteninae Simon, 1897, Calocteninae Simon, 1897 and Cteninae Keyserling, 1877 (Silva-Dávila 2003, Polotow and Brescovit 2014, Polotow et al. 2015). Cteninae is the largest subfamily in Ctenidae and can be distinguished from the remaining subfamilies by the embolus base with a large lobe in prolateral view and by the sclerotised median plate of the epigyne (Silva-Dávila 2003, Polotow and Brescovit 2012). In the subfamily Cteninae, *Bowie* Jäger, 2022 contains 107 species and 14 species groups and exhibits the highest diversity in Asia (101 spp.) (Chu et al. 2022, Jäger 2022, Lu et al. 2022a, World Spider Catalog 2022). Both *Amauropelma* Raven, Stumkat & Gray, 2001 and *Anahita* Karsch, 1879 are smaller genera, also belong to the subfamily Cteninae and currently contain 25 and 33 known species, respectively: nine *Amauropelma* species of which are known from Asia and 17 species of *Anahita* are known from Asia (Polotow and Brescovit 2014, Chu et al. 2022, Lu et al. 2022a, World Spider Catalog 2022).

Recently, a large number of new spider species have been reported from Asia, by combining morphological and molecular identification methods, especially from China (e.g. Li et al. 2021, Yao et al. 2021, Liu et al. 2022, Lu et al. 2022b). In this paper, based on the results of the morphology and DNA barcoding, nine new species of *Amauropelma*, *Anahita* and *Bowie* are described from Asia. This brings the total number of Ctenidae to 150 species in Asia, of which 139 species belong to Cteninae (Silva-Dávila 2003, Marusik et al. 2012, Polotow and Brescovit 2014, Jäger 2022, Lu et al. 2022a, World Spider Catalog 2022).

## Materials and methods

All specimens were preserved in 80% ethanol. Epigynes were cleared in trypsin enzyme solution to dissolve non-chitinous tissues. Specimens were examined under a LEICA M205 C stereomicroscope. Photomicroscopy images were taken with an Olympus C7070 zoom digital camera (7.1 megapixels). Photos were stacked with Helicon Focus® (Version 7.6.1) or Zerene Stacker® (Version 1.04) and processed in Adobe Photoshop CC2019®. All measurements are in millimetres (mm) and were obtained with an Olympus SZX16 stereomicroscope with a Zongyuan CCD industrial camera. Total length does not include the chelicerae. Palp and leg measurements are shown as: total length (femur, patella, tibia, metatarsus, tarsus). Leg segments were measured on their dorsal side. The distribution map was generated with ArcGIS 10.2 (ESRI Incorporated Company). References to figures in the cited papers are listed in lowercase (fig. or figs); figures from this paper are noted with a capital letter (Fig.). The type material is deposited in the Institute of Zoology, Chinese Academy of Sciences (IZCAS) in Beijing, China.

Size classes are used according to Jäger (2012), total lengths: small (< 10 mm), medium (10–20 mm), large (20–30 mm), very large (> 30 mm). Palp and leg claw dentition is given according to terminology in Jäger (2012). Arising points of the embolus, tegular apophysis and conductor in male palps are given as clock-positions of the left palp in ventral view. Spination pattern is given in two different formulae: in leg patellae and palp, the sum of all spines is listed for the prolateral, dorsal, retrolateral, ventral sides; when ventral spines are absent, only three digits are listed (Davies 1994, Jäger 2012). In other leg segments, spine positions are given from proximal to distal on each side (prolateral, dorsal, retrolateral, ventral, if present) following Jäger (2012). Leg formula is given as order of legs according to their length (femur to tarsus measured) in Arabic numbers, for example, 4123. For cheliceral teeth, large and small teeth are separated by “+”, for example, 4 + 1, meaning 4 large and 1 small teeth.

Terminology and taxonomic descriptions follow Jäger (2012) and Jäger (2022). The following abbreviations are used in the descriptions: ALE = anterior lateral eye, AME = anterior median eye, AW = anterior width of prosoma, d = dorsal, OL = opisthosoma length, OW = opisthosoma width, p = prolateral, PL = length of dorsal shield of prosoma, PLE = posterior lateral eye, PME = posterior median eye, PW = width of dorsal shield of prosoma, r = retrolateral, RTA = retrolateral tibial apophysis, v = ventral, I–IV = legs I to IV.

Total genomic DNA was extracted from legs of a single specimen. The DNA barcodes of all the species in this study were obtained, except for *B.vinhphuc* sp. n. A partial fragment of the mitochondrial cytochrome oxidase subunit I (COI) gene was amplified and sequenced, using the following primers: forward: LCO1490 (5’-CWACAAAYCATARRGATATTGG-3’) and reverse: HCO2198 (5'-TAAACTTCAGGGTGACCAAAAAATCA-3') (Folmer et al. 1994), except for *Am.phangnga* sp. n., using the following primers: forward: LCO1628 (5’-ATAATGTAATTGTTACTGCTCATGC-3’) and reverse: HCO2198 (5'-TAAACTTCAGGGTGACCAAAAAATCA-3') (Vandergast et al. 2004). COI p-distance is computed with MEGA 5 (Tamura et al. 2011). For additional information on extraction, amplification and sequencing procedures, see Yao et al. (2016). The sequences are deposited in GenBank.

## Taxon treatments

### Amauropelma krabi

S. Li & Yao sp. n.

C5E9E60C-A668-5AE7-A9FF-89D05F8A8027

57EA8144-E44C-4F59-8B3A-0D16719CB5D5

#### Materials

- **Type status:** Holotype. **Occurrence:** recordedBy: Z. Chen; individualCount: 1; sex: female; lifeStage: adult; **Taxon:** order: Araneae; family: Ctenidae; genus: Amauropelma; **Location:** country: Thailand; stateProvince: Krabi; verbatimLocality: Ao Luk District, Klang Cave; verbatimElevation: 36 m a.s.l.; verbatimLatitude: 8°20.268'N; verbatimLongitude: 98°44.707'E; **Event:** year: 2015; month: 10; day: 12; **Record Level:** institutionCode: IZCAS-Ar 43530

#### Description

**Male**

Unknown.

**Female** (IZCAS-Ar 43530): PL 3.3, PW 2.4, AW 1.6, OL 3.1, OW 1.6. Eye diameters and interdistances: AME 0.10, ALE 0.13, PME 0.11, PLE 0.11, AME–AME 0.04, AME–ALE 0.11, PME–PME 0.06, PME–PLE 0.24, AME–PME 0.06, ALE–PLE 0.08, clypeus AME 0.12, clypeus ALE 0.17. Palp and leg measurements: palp 3.8 (1.3, 0.7, 0.8, -, 1.0), I 10.3 (2.8, 1.6, 2.8, 2.1, 1.0), II 9.2 (2.4, 1.4, 2.4, 2.0, 1.0), III 9.0 (2.4, 1.3, 2.0, 2.2, 1.1), IV 12.2 (3.1, 1.4, 2.9, 3.5, 1.3). Leg formula 4123. Spination of palp and legs: palp 130, 100, 1111, 1212; femora I p002, d111, r010, II p010, d111, r010, III p111, d111, r012, IV p002, d111, r102; patellae I–IV 001; tibiae I–II v22222, III p11, d111, r11, v222, IV p111, d11, r11, v222; metatarsi I–II v222, III p112, d010, r112, v222, IV p112, r112, v222. Chelicerae with 3 promarginal, 4 + 1 retromarginal teeth, without denticles. Retromargin of chelicerae close to fang base without bristle. Tarsi and metatarsi without scopula. Claw tufts arising separately, but intermingle with each other distally. Palpal claw with 3 secondary teeth, leg claws I–II with 3, III with 2 and IV with 3 secondary teeth. Position of tarsal organ: I 0.76, II 0.72, III 0.68.

Copulatory organ (Fig. 2a, b, Fig. 3a and b). Epigynal plate width/length: 9.8/6.5; anterior width/posterior width: 9.8/7.5; heart-shaped and with a mating plug, the anterior part with a pair of pointed apophyses ventrally. Lateral teeth pointing postero-medially. Internal duct system with small oval spermathecae not fully visible, separated from each other by more than their diameter; fertilisation ducts elongate and laminar, pointing postero-medially.

Colour (Fig. 2c and d). Reddish-brown to yellowish without patterns. Dorsal prosoma slightly reddish-brown to yellowish, with eyes marked with black rings, fovea distinct, reddish-brown. Chelicerae reddish-brown. Sternum, ventral coxae, labium yellowish-brown without patterns. Gnathocoxae yellowish-brown with lighter distal lips. Legs yellowish-brown. Opisthosoma yellowish. Spinnerets yellowish.

#### Diagnosis

Small Ctenidae (total length female 6.4). The new species can be distinguished from all known congeners by the median plate roughly heart-shaped and with a mating plug (Fig. 2a and Fig. 3a), by the anterior part of median plate with a pair of pointed apophyses ventrally (arrowed in Fig. 2a, arrowed in Fig. 3a), by the internal duct system with small oval spermathecae not fully visible (Fig. 2b and Fig. 3b) and by the fertilisation ducts which are elongate and laminar, almost twice as long as the spermathecae (Fig. 2b and Fig. 3b).

#### Etymology

The specific name refers to the type locality and is a noun in apposition.

#### Distribution

**Figure 1.**
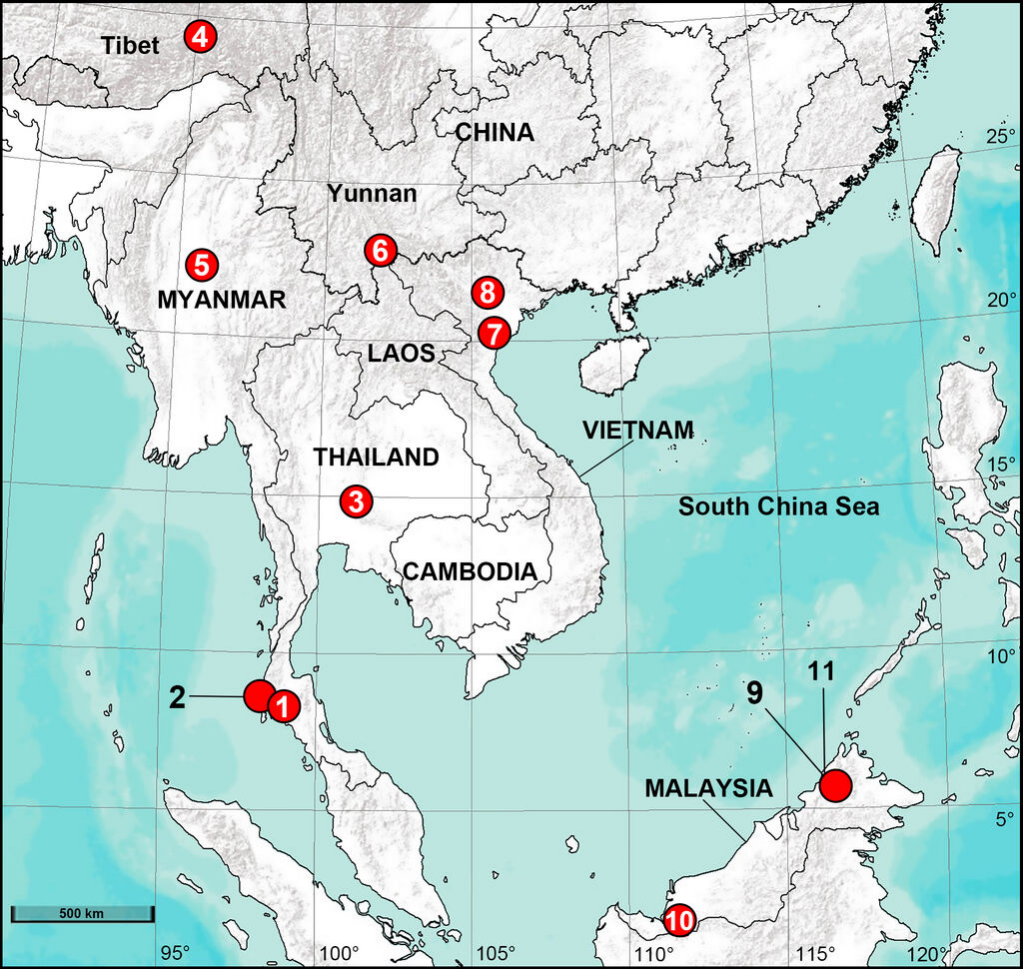
Distribution records of ctenid spiders from Asia in this study. 1. *Amauropelmakrabi* sp. n.; 2. *Am.phangnga* sp. n.; 3. *Am.saraburi* sp. n.; 4. *Anahitamedog* sp. n.; 5. *An.popa*; 6. *Bowiefascination*; 7. *B.ninhbinh* sp. n.; 8. *B.vinhphuc* sp. n.; 9. *B.borneo* sp. n.; 10. *B.engkilili* sp. n.; 11. *B.sabah* sp. n.

**Figure 2.**
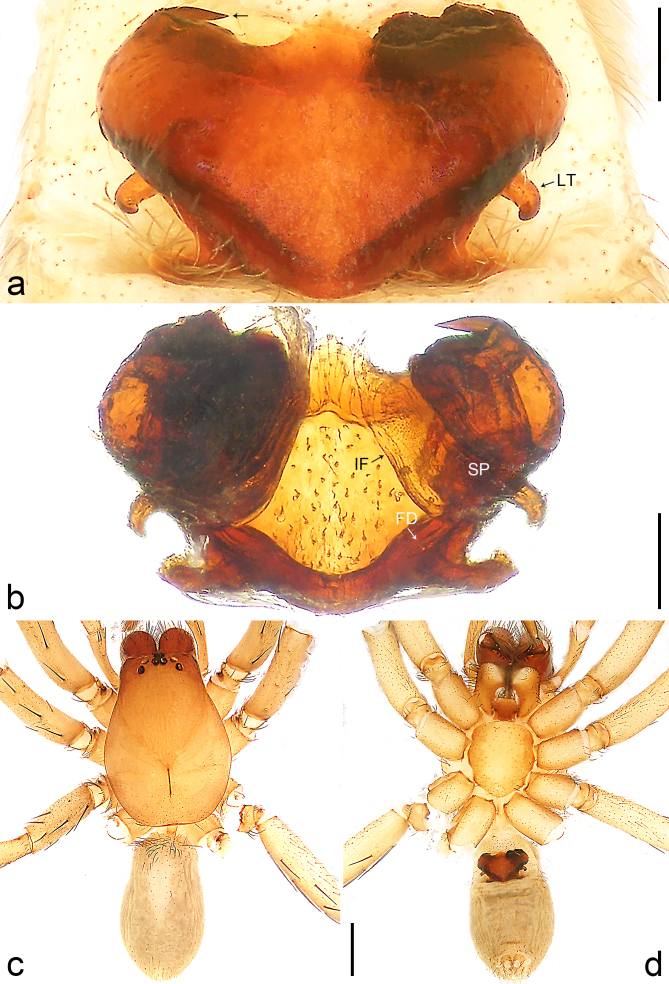
*Amauropelmakrabi* sp. n., holotype female. **a**: Epigyne, ventral view, arrow points at pointed apophysis; **b**: Vulva, dorsal view; **c**: Habitus, dorsal view; **d**: Habitus, ventral view. FD = fertilisation duct, IF = internal fold, LT = lateral teeth, SP = spermathecae. Scale bars: 0.2 mm (**a**, **b**), 1.0 mm (**c**, **d**).

**Figure 3.**
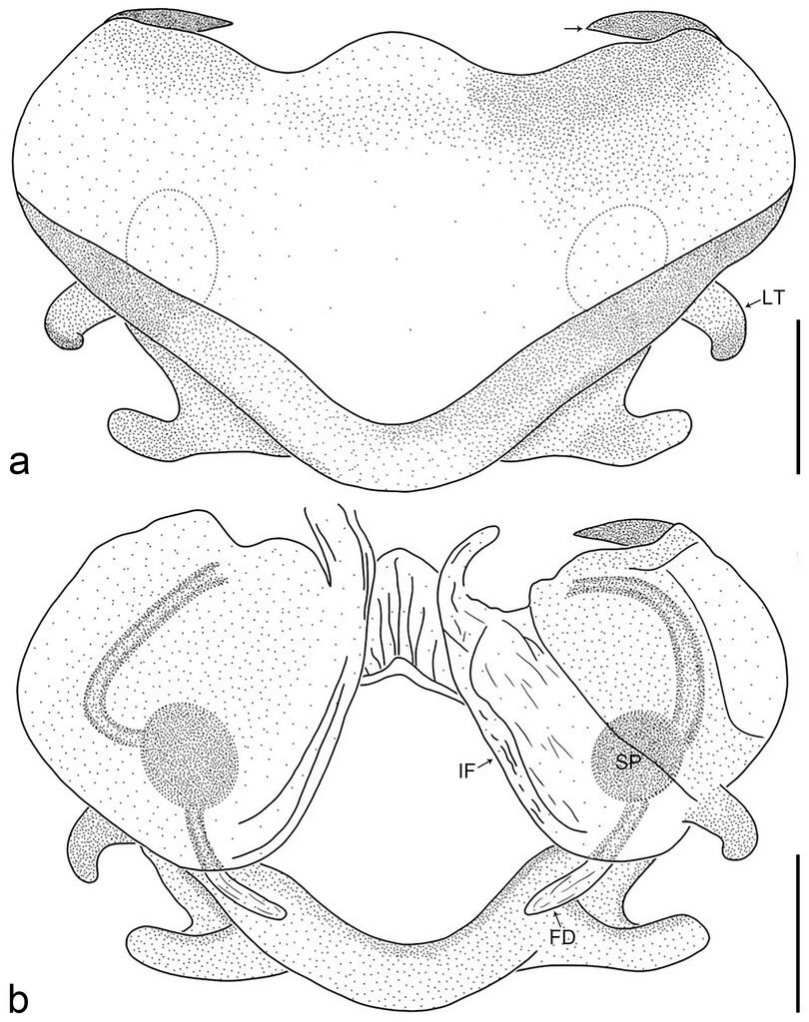
*Amauropelmakrabi* sp. n., holotype female. **a**: Epigyne, ventral view, arrow points at pointed apophysis; **b**: Vulva, dorsal view. FD = fertilisation duct, IF = internal fold, LT = lateral teeth, SP = spermathecae. Scale bars: 0.2 mm (**a**, **b**).

Thailand (Krabi, type locality; Fig. 1).

#### DNA Barcode

Female (IZCAS-Ar 43530):

TGTTTGGAGCTTGAGCTGCTATAGCAGGAACTGGAATAAGAGTGTTGATTCGAATAGAGTTAGGTCATCCTGGTAGATTGTTAGGAGATGATCATTTATATAATGTTATTGTAACTGCTCATGCTTTTGTAATGATTTTTTTTATAGTAATACCAATTTTGATTGGTGGATTTGGAAATTGATTAGTTCCGTTGAGATTGGAGCACCTGATATATCATTTCCTCGAATAAATAATTTGTCGTTTTGATTACTACCTCCTTCTTTATTTTTATTAATAATATCATCAATAGTAGAAATAGGTGTTGGAGCGGGATGAACTGTTTATCCTCCTTTAGCATCTAGTATTGGGCATATAGGAAGATCTATAGATTTTGCTATTTTTTCTCTTCATTTGGCTGGAGCTTCTTCTATTATAGGAGCAGTAAATTTTATTTCTACTATTATTAATATACGGTTGTATGGAATGAGTATAGAAAAGGTTCCTTTGTTTGTGTGGTCTGTTTTTATTACTGCTATTTTGTTATTATTGTCGTTACCTGTGTTAGCAGGTGCTATTACTATATTATTGACTGATCGAAATTTTAATACTTCTTTTTTTGACCCTGCGGGAGGGGGAGATCCTATTTTGTTTCAACATTTATTTTGATTTTTTG (GenBank accession number OP561682).

### Amauropelma phangnga

S. Li & Yao sp. n.

EB19D14B-F9BF-5E7F-AB6D-BB26A0112DEA

AA485B79-DD09-4AEF-98B8-8E12698B19F4

#### Materials

- **Type status:** Holotype. **Occurrence:** recordedBy: Z. Chen; individualCount: 1; sex: male; lifeStage: adult; **Taxon:** order: Araneae; family: Ctenidae; genus: Amauropelma; **Location:** country: Thailand; stateProvince: Phang Nga; verbatimLocality: Mueang District, Tapan Cave; verbatimElevation: 35 m a.s.l.; verbatimLatitude: 8°27.305'N; verbatimLongitude: 98°31.690'E; **Event:** year: 2015; month: 10; day: 10; **Record Level:** institutionCode: IZCAS-Ar 43531
- **Type status:** Paratype. **Occurrence:** recordedBy: Z. Chen; individualCount: 1; sex: male; lifeStage: adult; **Taxon:** order: Araneae; family: Ctenidae; genus: Amauropelma; **Location:** country: Thailand; stateProvince: Phang Nga; verbatimLocality: Mueang District, Tapan Cave; verbatimElevation: 35 m a.s.l.; verbatimLatitude: 8°27.305'N; verbatimLongitude: 98°31.690'E; **Event:** year: 2015; month: 10; day: 10; **Record Level:** institutionCode: IZCAS-Ar 43532

#### Description

**Male** (IZCAS-Ar 43531): PL 3.3, PW 2.8, AW 1.2, OL 3.0, OW 1.9. Eye diameters and interdistances: AME 0.09, ALE 0.12, PME 0.10, PLE 0.10, AME–AME 0.04, AME–ALE 0.13, PME–PME 0.08, PME–PLE 0.21, AME–PME 0.05, ALE–PLE 0.08, clypeus AME 0.17, clypeus ALE 0.22. Palp and leg measurements: palp 3.8 (0.9, 0.6, 0.8, -, 1.5), I 13.5 (3.3, 1.6, 3.7, 3.2, 1.7), II 11.6 (3.1, 1.6, 3.0, 2.6, 1.3), III 10.9 (2.9, 1.4, 2.7, 2.6, 1.3), IV 14.8 (3.5, 1.5, 3.9, 4.2, 1.7). Leg formula 4123. Spination of palp and legs: palp 131, 100, 1101; femora I p021, d211, r112, II–III p012, d111, r012, IV p102, d111, r012; patellae I–IV 001; tibiae I p010, v22222, II p100, r100, v22222, III p11, d111, r11, v222, IV p11, d11, r11, v222; metatarsi I v222, II p112, r010, v222, III p112, d010, r112, v222, IV p112, r112, v2222. Chelicerae with 3 promarginal, 4 retromarginal teeth, without denticles. Retromargin of chelicerae close to fang base without bristle. Tarsi and metatarsi without scopula. Claw tufts arising separately, but intermingle with each other distally. Leg claws I with 7 and II with 6 secondary teeth. Position of tarsal organ: I 1.37, II 0.92, III 0.85.

**Figure 4.**
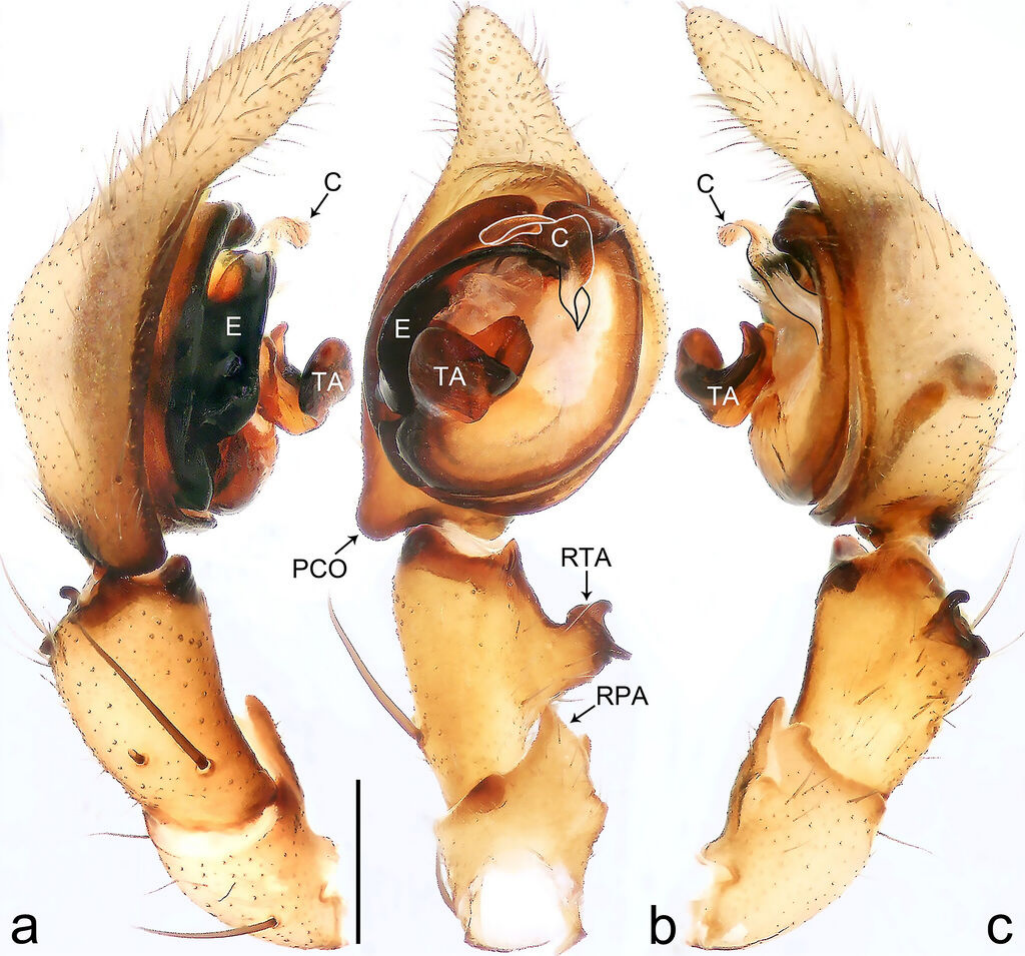
*Amauropelmaphangnga* sp. n., palp, holotype. **a**: Prolateral view; **b**: Ventral view; **c**: Retrolateral view. C = conductor, E = embolus, PCO = prolatero-proximal cymbial outgrowth, RPA = retrolateral patellar apophysis, RTA = retrolateral tibial apophysis, TA = tegular apophysis. Scale bar: 0.5 mm (**a–c**).

Palp (Fig. 4a–c). Patella with distinct retrolateral apophysis. RTA protruding at an almost right angle from tibia in ventral view, with broad base and two short apices, both dorso-distad. Cymbium tip conical, with prolatero-proximal outgrowth. Embolus (Fig. 9a) arising at 8 o’clock position, its tip with an extension. Conductor arising at 3 o’clock position, long and laminar, running around tegulum anti-clockwise, its tip situated subdistally. Tegular apophysis arising subcentrally, strongly concave on ventral side and distinctly excavated on prolateral side.

**Figure 5.**
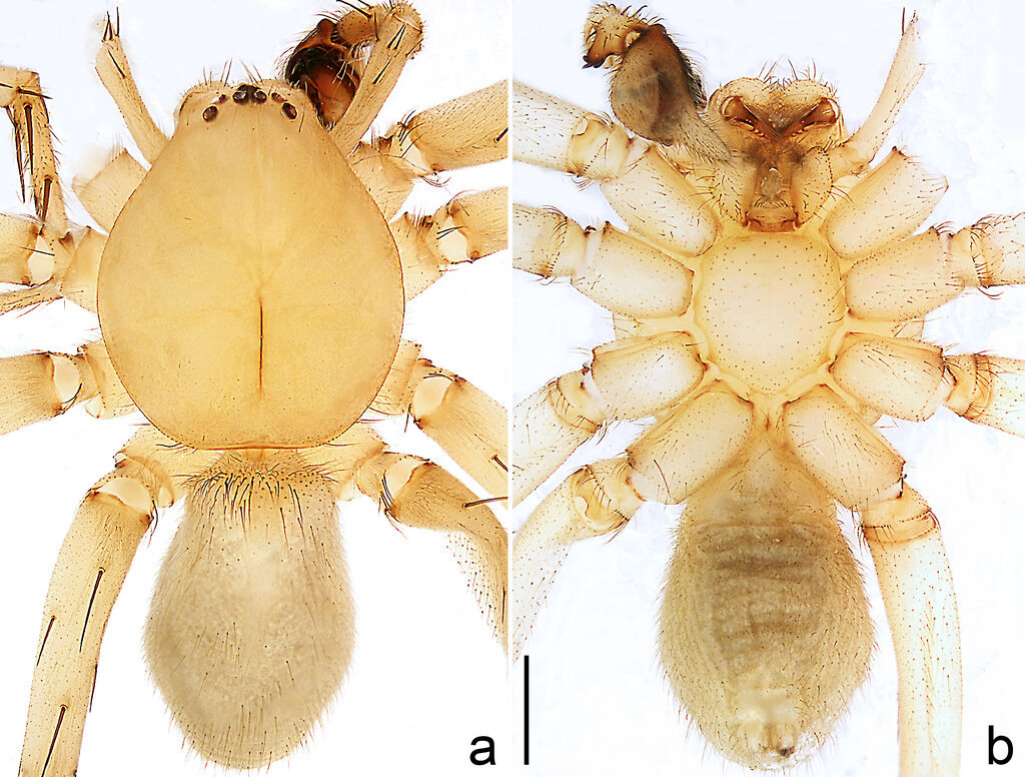
*Amauropelmaphangnga* sp. n., habitus, holotype male. **a**: Dorsal view; **b**: Ventral view. Scale bar: 1.0 mm (**a**, **b**).

Colour (Fig. 5a and b). Yellowish-brown. Dorsal prosoma with eyes marked with black rings, fovea distinct, reddish-brown. Ventral opisthosoma grey.

**Female**

Unknown.

**Variation**: Paratype male (IZCAS-Ar 43532): PL 3.4, OL 2.4.

#### Diagnosis

Small Ctenidae (total length male 5.8–6.3). The new species can be distinguished from all known congeners by the embolus tip with an extension (Fig. 4b and Fig. 9a), by the tegular apophysis strongly concave on ventral side and distinctly excavated on prolateral side (Fig. 4a and b), by the conductor arising at 3 o’clock position, long and laminar, running around tegulum anti-clockwise, its tip situated subdistally (Fig. 4b), by the RTA protruding at an almost right angle from tibia in ventral view, with broad base and two short apices, both dorso-distad (Fig. 4b) and by the patella with distinct retrolateral apophysis, pointing anteriorly (Fig. 4b). This species can also be distinguished from *Am.krabi* sp. n. by the COI p-distance 0.134 between them.

#### Etymology

The specific name refers to the type locality and is a noun in apposition.

#### Distribution

Thailand (Phang Nga, type locality; Fig. 1).

#### DNA Barcode

Male (IZCAS-Ar 43532):

GGTGGGTTCGGAAATTGATTGGTTCCTTTGATGTTAGGAGCTCCTGATATATCATTTCCTCGTATAAATAATTTGTCTTTTTGGTTACTTCCTCCTTCTTTATTTTTGTTATTAATATCTTCTATGGTGGAAATAGGAGTGGGAGCAGGATGAACTGTCTATCCTCCTTTAGCTTCTAGAATAGGGCATGTGGGAAGATCAATAGATTTTGCGATTTTTTCTCTTCATTTAGCTGGAGTTTCTTCTATTATGGGAGCGGTTAATTTTATTTCTACTATTATTAATATGCGATTATATGGAATAACTATAGAAAAGGTTCCTTTATTCGTTTGATCAGTTTTTATTACTGCAGTTTTGTTGTTGTTATCATTACCTGTGTTAGCAGGTGCTATTACTATATTATTGACAGATCGAAATTTTAATACTTCTTTTTTTGATCCTGCAGGGGGTGGAGATCCAATTTTATTTCAACATTTATTCTGATTTTTTGGTCACCCTGGAAAGTTTAA (GenBank accession number OP718556).

### Amauropelma saraburi

S. Li & Yao sp. n.

7FEEE19B-A404-5A83-8C1B-2140ABF97D1B

3009CDF3-0EF9-4FE6-A1EF-C3C0EF376C94

#### Materials

- **Type status:** Holotype. **Occurrence:** recordedBy: Z. Chen; individualCount: 1; sex: male; lifeStage: adult; **Taxon:** order: Araneae; family: Ctenidae; genus: Amauropelma; **Location:** country: Thailand; stateProvince: Saraburi; verbatimLocality: Kaeng Khoi District, Song Khon Village, Tham Bo Pla Cave; verbatimElevation: 73 m a.s.l.; verbatimLatitude: 14°39.625'N; verbatimLongitude: 100°58.115'E; **Event:** year: 2014; month: 10; day: 20; **Record Level:** institutionCode: IZCAS-Ar 43533
- **Type status:** Paratype. **Occurrence:** recordedBy: Z. Chen; individualCount: 1; sex: female; lifeStage: adult; **Taxon:** order: Araneae; family: Ctenidae; genus: Amauropelma; **Location:** country: Thailand; stateProvince: Saraburi; verbatimLocality: Kaeng Khoi District, Song Khon Village, Tham Bo Pla Cave; verbatimElevation: 73 m a.s.l.; verbatimLatitude: 14°39.625'N; verbatimLongitude: 100°58.115'E; **Event:** year: 2014; month: 10; day: 20; **Record Level:** institutionCode: IZCAS-Ar 43534
- **Type status:** Paratype. **Occurrence:** recordedBy: Z. Chen; individualCount: 1; sex: female; lifeStage: adult; **Taxon:** order: Araneae; family: Ctenidae; genus: Amauropelma; **Location:** country: Thailand; stateProvince: Saraburi; verbatimLocality: Kaeng Khoi District, Song Khon Village, Tham Bo Pla Cave; verbatimElevation: 73 m a.s.l.; verbatimLatitude: 14°39.625'N; verbatimLongitude: 100°58.115'E; **Event:** year: 2014; month: 10; day: 20; **Record Level:** institutionCode: IZCAS-Ar 43535

#### Description

**Male** (IZCAS-Ar 43533): PL 4.5, PW 3.7, AW 1.6, OL 3.2, OW 2.1. Eye diameters and interdistances: AME 0.12, ALE 0.14, PME 0.14, PLE 0.14, AME–AME 0.07, AME–ALE 0.16, PME–PME 0.08, PME–PLE 0.26, AME–PME 0.07, ALE–PLE 0.13, clypeus AME 0.16, clypeus ALE 0.28. Palp and leg measurements: palp 5.2 (1.8, 0.8, 0.9, -, 1.7), I - (4.5, 2.0, 4.5, 4.0, -), II 15.2 (3.8, 2.1, 3.8, 3.7, 1.8), III 14.3 (3.6, 1.8, 3.4, 3.7, 1.8), IV 19.4 (4.9, 2.0, 4.6, 5.6, 2.3). Leg formula 4123. Spination of palp and legs: palp 131, 100, 210; femora I p112, d111, r111, II p211, d111, r211, III p112, d111, r112, IV p112, d111, r002; patellae I 001, II–IV 101; tibiae I p010, r110, v22222, II p100, r100, v22222, III–IV p11, d111, r11, v222; metatarsi I v222, II p110, r110, v222, III p112, d010, r112, v222, IV p112, d010, r112, v2222. Chelicerae with 3 promarginal, 4 retromarginal teeth, without denticles. Retromargin of chelicerae close to fang base without bristle. Claw tufts arising separately, but intermingle with each other distally. Leg claws II with 1 and III–IV with 2 secondary teeth. Position of tarsal organ: IV 1.58.

**Figure 6.**
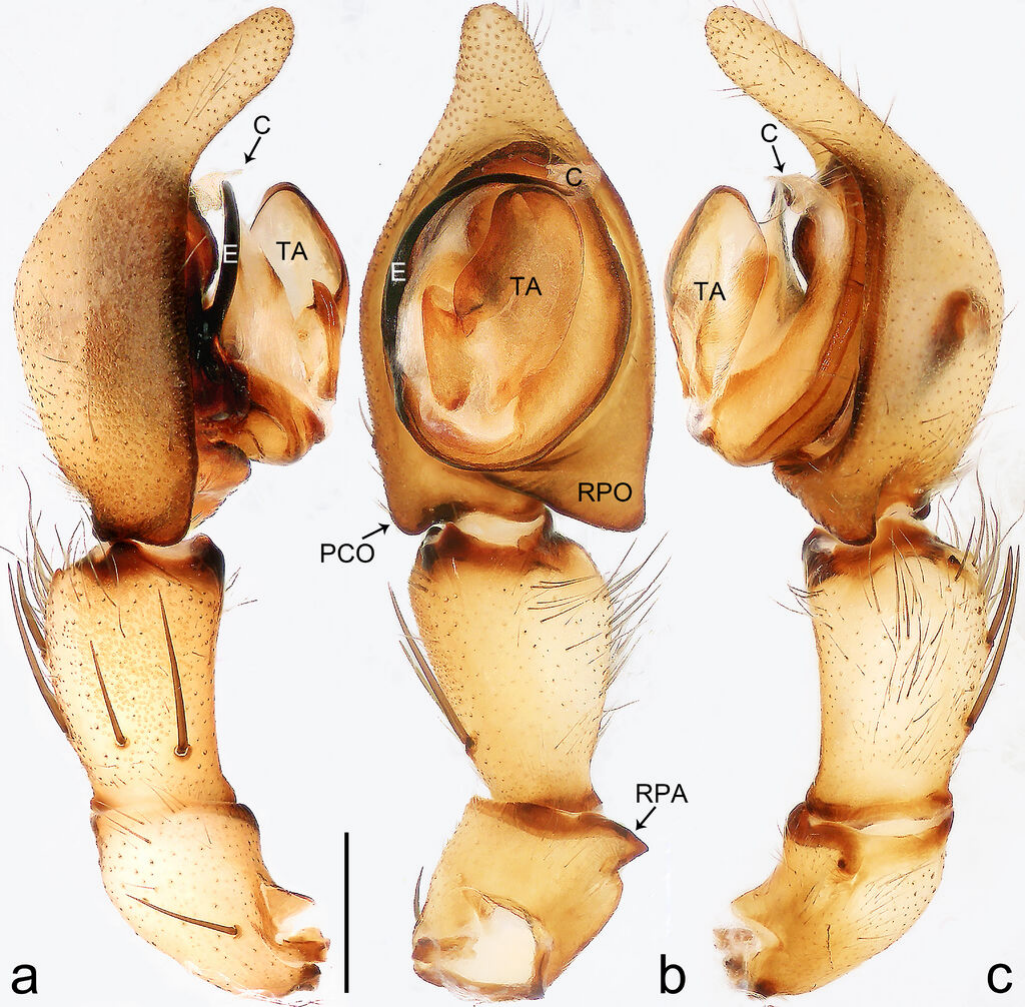
*Amauropelmasaraburi* sp. n., flipped right palp, holotype. **a**: Prolateral view; **b**: Ventral view; **c**: Retrolateral view. C = conductor, E = embolus, PCO = prolatero-proximal cymbial outgrowth, RPA = retrolateral patellar apophysis, RPO = retro-proximal cymbial outgrowth, TA = tegular apophysis. Scale bar: 0.5 mm (**a–c**).

Palp (Fig. 6a–c). Patella with distinct retrolateral apophysis. Cymbium tip conical, with prolatero-proximal outgrowth and retro-proximal outgrowth. Embolus (Fig. 9b) slender, arising at 8.30 o’clock position. Conductor arising at 1 o’clock position. Tegular apophysis large and longitudinally elongated, with excavation on prolateral side.

**Figure 7.**
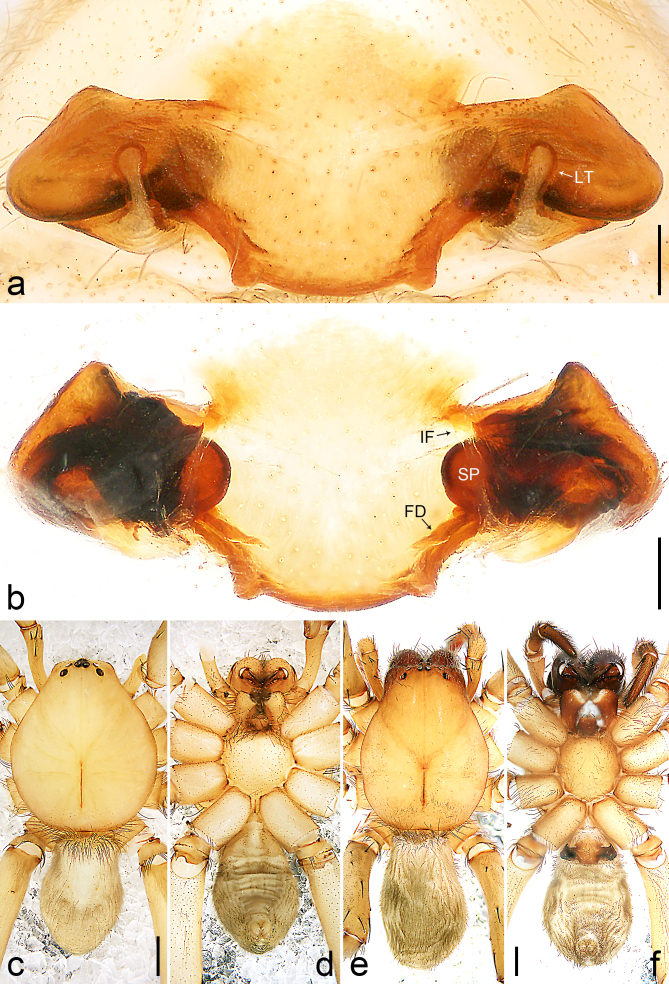
*Amauropelmasaraburi* sp. n. **a**: Paratype female, epigyne, ventral view; **b**: Same, vulva, dorsal view; **c**: Holotype male, habitus, dorsal view; **d**: Same, habitus, ventral view; **e**: Paratype female, habitus, dorsal view; **f**: Same, habitus, ventral view. FD = fertilisation duct, IF = internal fold, LT = lateral teeth, SP = spermathecae. Scale bars: 0.2 mm (**a**, **b**), 1.0 mm (**c–f**).

Colour (Fig. 7c and d). Yellowish-brown. Dorsal prosoma yellowish with eyes marked with black rings, fovea distinct, brown. Sternum, ventral coxae, labium and gnathocoxae yellowish without patterns. Chelicerae brown. Legs yellowish. Dorsal opisthosoma yellowish without patterns. Lateral and ventral opisthosoma grey without patterns. Spinnerets grey.

**Female** (IZCAS-Ar 43534): PL 5.6, PW 4.4, AW 2.8, OL 4.9, OW 3.1. Eye diameters and interdistances: AME 0.14, ALE 0.20, PME 0.15, PLE 0.16, AME–AME 0.12, AME–ALE 0.32, PME–PME 0.16, PME–PLE 0.53, AME–PME 0.08, ALE–PLE 0.20, clypeus AME 0.13, clypeus ALE 0.21. Palp and leg measurements: palp 6.0 (1.7, 1.2, 1.4, -, 1.7), I 19.3 (4.6, 2.7, 5.6, 4.2, 2.2), II 18.3 (4.9, 2.5, 5.0, 4.0, 1.9), III 17.0 (4.6, 2.2, 4.1, 4.3, 1.8), IV 23.0 (5.8, 2.4, 5.5, 6.6, 2.7). Leg formula 4123. Spination of palp and legs: palp 131, 100, 1111, 2112; femora I p021, d111, r111, II–III p112, d111, r112, IV p112, d111, r002; patellae I–II 000, III–IV 101; tibiae I –II v22222, III–IV p11, d111, r11, v222; metatarsi I–II v222, III–IV p112, d010, r112, v222. Chelicerae with 3 promarginal, 4 retromarginal teeth, without denticles. Retromargin of chelicerae close to fang base without bristle. Sparse scopula restricted almost entirely to tarsi, only metatarsi I–II with sparse scopula hairs. Claw tufts arising separately, but intermingle with each other distally. Palpal claw with 3 secondary teeth, leg claws I with 3, II–III with 2 secondary teeth.

**Figure 8.**
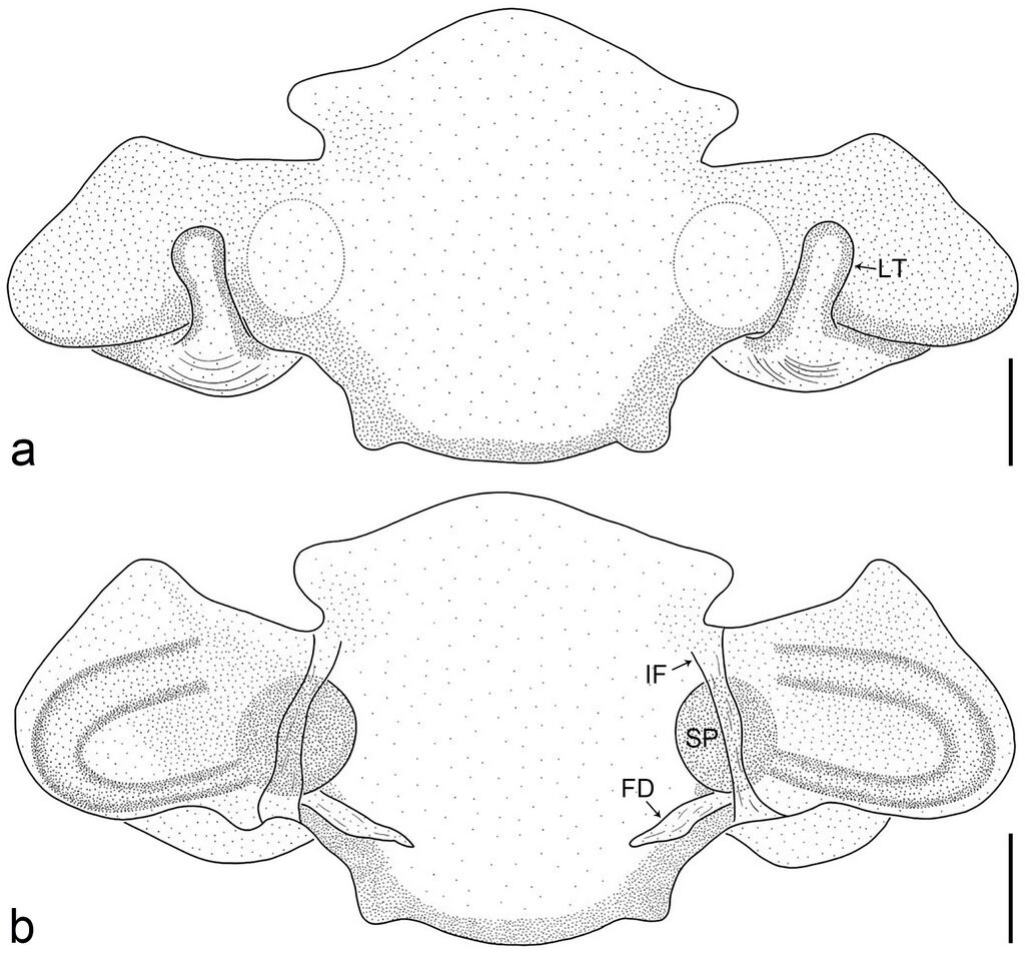
*Amauropelmasaraburi* sp. n., paratype female. **a**: Epigyne, ventral view; **b**: Vulva, dorsal view. FD = fertilisation duct, IF = internal fold, LT = lateral teeth, SP = spermathecae. Scale bars: 0.2 mm (**a**, **b**).

Copulatory organ (Fig. 7a, b, Fig. 8a and b). Epigynal plate width/length: 19.5/8.6; anterior width/posterior width: 15/6.3; with lateral wings and posterior part with distinct lateral margins and two distinct tubercles. Lateral teeth pointing anteriorly. Internal duct system with round spermathecae not fully visible and spermathecae separated from each other by more than their diameter; fertilisation ducts elongate and laminar, pointing postero-medially.

Colour (Fig. 7e and f). Reddish-brown to yellowish. Dorsal prosoma slightly reddish-brown with eyes marked with black rings, fovea distinct, reddish-brown. Sternum and ventral coxae yellowish without patterns; labium and gnathocoxae reddish-brown without patterns. Chelicerae reddish-brown. Legs yellowish-brown. Dorsal and lateral opisthosoma grey without patterns. Ventral opisthosoma yellowish without patterns. Spinnerets yellowish.

**Variation**: Second paratype female (IZCAS-Ar 43535): PL 4.6, OL 4.7.

#### Diagnosis

Small to medium-sized Ctenidae (total length male 7.7, female 9.3–10.5). The new species can be distinguished from all known congeners by the embolus slender (Fig. 6b and Fig. 9b), by the tegular apophysis large and longitudinally elongated, with excavation on prolateral side (Fig. 6a–c), by the epigynal field wider than long, with lateral wings and distinct lateral margins (Fig. 7a and Fig. 8a) and by the lateral teeth pointing anteriorly (Fig. 7a and Fig. 8a).

#### Etymology

The specific name refers to the type locality and is a noun in apposition.

#### Distribution

Thailand (Saraburi, type locality; Fig. 1).

#### DNA Barcode

Male (IZCAS-Ar 43533):TGTTTGGAGCTAGATCTGCTATAGCGGGAACGGCAATAAGAGTTTTAATTCGTATGGAATTAGGAAATTCTGGAAGATTATTAGGGGATGATCATTTATATAATGTAATTGTGACAGCTCATGCTTTTATTATGATTTTTTTTATAGTAATACCGATTTTGATTGGTGGTTTTGGAAATTGATTAGTGCCTTTAATGTTAGGAGCTCCTGATATATCTTTTCCTCGGATGAATAATTTGTCTTTTTGATTACTTCCACCTTCTTTGTTTTTATTATTCATATCTTCTATGGTGGAAATGGGTGTAGGAGCTGGATGAACTGTTTATCCACCTTTGGCTTCTAGAATTGGTCATGCTGGAAGATCTATGGATTTTGCTATTTTTTCTTTACATTTAGCTGGGGCTTCTTCAATTATAGGAGCGGTGAATTTTATTTCTACTATTATTAATATACGATTATCTGGAATAAGAATGGAGAAGGTTCCATTATTTGTTTGATCTGTTCTTATTACTGCAATTTTATTATTATTATCTTTGCCGGTATTAGCTGGTGCTATTACTATATTGTTGACTGATCGAAATTTTAATACTTCTTTTTTTGATCCGGCTGGGGGAGGGGATCCTATTTTATTTCAACATTTATTTTGATTTTTTG (GenBank accession number OP572100).

Female (IZCAS-Ar 43534):TGTTTGGAGCTTGATCTGCTATAGCGGGAACGGCAATAAGAGTTTTAATTCGTATGGAATTAGGAAATTCTGGAAGATTATTAGGGGATGATCATTTATATAATGTAATTGTGACAGCTCATGCTTTTATTATGATTTTTTTTATAGTAATACCGATTTTGATTGGTGGTTTTGGAAATTGATTAGTGCCTTTAATGTTAGGAGCTCCTGATATATCTTTTCCTCGGATGAATAATTTGTCTTTTTGATTACTTCCACCTTCTTTGTTTTTATTATTCATATCTTCTATGGTGGAAATGGGTGTAGGAGCTGGATGAACTGTTTATCCACCTTTGGCTTCTAGAATTGGTCATGCTGGAAGATCTATGGATTTTGCTATTTTTTCTTTACATTTAGCTGGGGCTTCTTCAATTATAGGAGCGGTGAATTTTATTTCTACTATTATTAATATACGATTATCTGGAATAAGAATGGAGAAGGTTCCATTATTTGTTTGATCTGTTCTTATTACTGCAATTTTATTATTATTATCTTTGCCGGTATTAGCTGGTGCTATTACTATATTGTTGACTGATCGAAATTTTAATACTTCTTTTTTTGATCCGGCTGGGGGAGGGGATCCTATTTTATTTCAACATTTATTTTGATTTTTTG (GenBank accession number OP572099).

### Anahita medog

S. Li & Yao sp. n.

FF71E5C3-DA0C-58D9-9400-968831F657BF

C86093E4-F706-4CB0-B5C4-FA9DB913CD34

#### Materials

- **Type status:** Holotype. **Occurrence:** recordedBy: J. Wu; individualCount: 1; sex: male; lifeStage: adult; **Taxon:** order: Araneae; family: Ctenidae; genus: Anahita; **Location:** country: China; stateProvince: Tibet; municipality: Nyingchi; locality: Medog County; verbatimLocality: Baibung Town, near the Jiagagou Bridge; verbatimElevation: 805 m a.s.l.; verbatimLatitude: 29°15.067'N; verbatimLongitude: 95°11.717'E; **Event:** samplingProtocol: Collected by hand in leaf litter; year: 2016; month: 6; day: 18; **Record Level:** institutionCode: IZCAS-Ar 43536
- **Type status:** Paratype. **Occurrence:** recordedBy: J. Wu; individualCount: 1; sex: female; lifeStage: adult; **Taxon:** order: Araneae; family: Ctenidae; genus: Anahita; **Location:** country: China; stateProvince: Tibet; municipality: Nyingchi; locality: Medog County; verbatimLocality: Baibung Town, near the Jiagagou Bridge; verbatimElevation: 805 m a.s.l.; verbatimLatitude: 29°15.067'N; verbatimLongitude: 95°11.717'E; **Event:** samplingProtocol: Collected by hand in leaf litter; year: 2016; month: 6; day: 18; **Record Level:** institutionCode: IZCAS-Ar 43537

#### Description

**Male** (IZCAS-Ar 43536): PL 2.7, PW 2.3, AW 0.9, OL 2.4, OW 1.4. Eye diameters and interdistances: AME 0.13, ALE 0.10, PME 0.22, PLE 0.19, AME–AME 0.10, AME–ALE 0.22, PME–PME 0.16, PME–PLE 0.23, AME–PME 0.11, ALE–PLE 0.14, clypeus AME 0.10, clypeus ALE 0.37. Palp and leg measurements: palp 4.1 (1.5, 0.6, 0.8, -, 1.2), I missing, II 13.0 (3.6, 1.2, 3.7, 3.3, 1.2), III 10.9 (2.8, 1.1, 2.8, 3.0, 1.2), IV 16.0 (4.1, 1.1, 4.2, 5.1, 1.5). Leg formula 4123. Spination of palp and legs: palp 023, 000, 0211; femora II p112, d111, r012, III p112, d111, r112, IV p112, d111, r012; patellae II–IV 101; tibiae II p110, d101, r100, v22222, III p11, d111, r11, v222, IV p11, d111, r11, v22; metatarsi II p111, r111, v222, III p112, d010, r112, v222, IV p112, d010, r112, v2222. Chelicerae with 3 promarginal, 4 + 1 retromarginal teeth and with elongated patch of 6 tiny denticles along entire cheliceral furrow. Leg claws II with 9, III with 5 and IV with 7 secondary teeth. Position of tarsal organ: II 1.06, III 0.85, IV 1.14.

**Figure 10.**
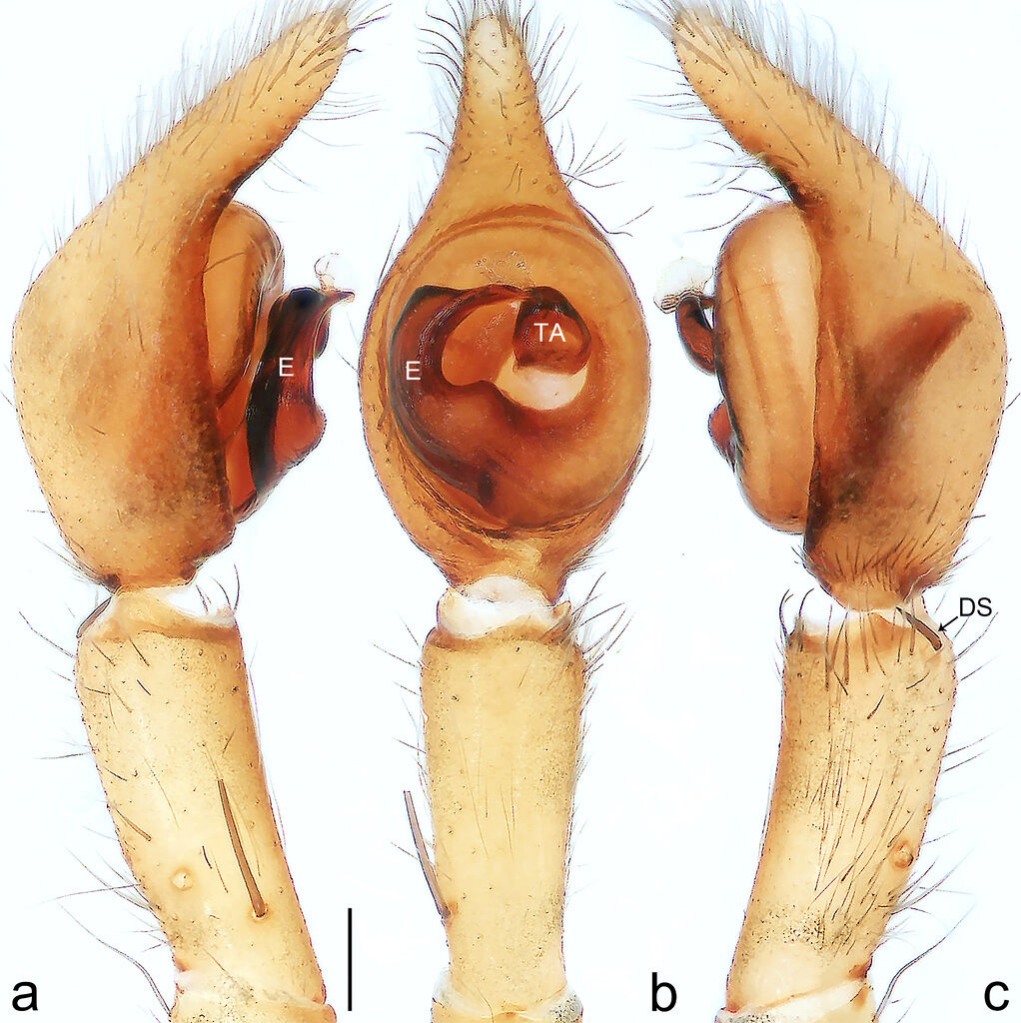
*Anahitamedog* sp. n., palp, holotype. **a**: Prolateral view; **b**: Ventral view; **c**: Retrolateral view. DS = distal retrolateral spine, E = embolus, TA = tegular apophysis. Scale bar: 0.2 mm (**a–c**).

Palp (Fig. 10a–c). Palpal tibia without RTA and intrasegmental sclerite, distally with retrolateral stout spine. Cymbium tip conical. Embolus (Fig. 14a) arising at 6.30 o’clock position, with wide base and narrow tip and a membranous apophysis apically. Conductor absent. Tegular apophysis arising nearly centrally from tegulum.

**Figure 11.**
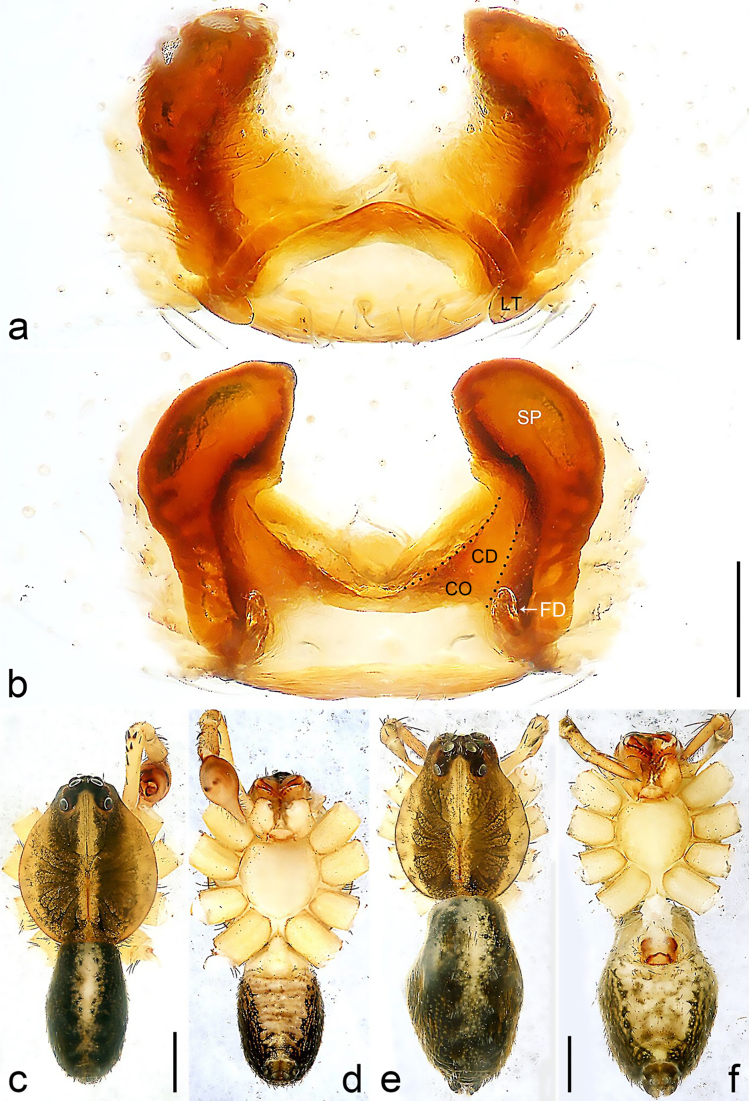
*Anahitamedog* sp. n. **a**: Paratype female, epigyne, ventral view; **b**: Same, vulva, dorsal view; **c**: Holotype male, habitus, dorsal view; **d**: Same, habitus, ventral view; **e**: Paratype female, habitus, dorsal view; **f**: Same, habitus, ventral view. CD = copulatory duct, CO = copulatory opening, FD = fertilisation duct, LT = lateral teeth, SP = spermathecae. Scale bars: 0.2 mm (**a**, **b**), 1.0 mm (**c–f**).

Colour (Fig. 11c and d). Black to yellowish. Dorsal prosoma with two parallel black lateral bands and distinctly marked fovea. Sternum, ventral coxae, labium and gnathocoxae yellowish. Chelicerae yellowish with longitudinal lines. Palps and legs yellowish. Dorsal opisthosoma black with light median band. Lateral opisthosoma spotted. Ventral opisthosoma yellowish with lateral black patterns. Spinnerets dark.

**Female** (IZCAS-Ar 43537): PL 2.9, PW 2.4, AW 1.2, OL 3.5, OW 2.0. Eye diameters and interdistances: AME 0.13, ALE 0.11, PME 0.19, PLE 0.18, AME–AME 0.13, AME–ALE 0.26, PME–PME 0.21, PME–PLE 0.24, AME–PME 0.15, ALE–PLE 0.17, clypeus AME 0.10, clypeus ALE 0.38. Palp and leg measurements: palp 3.2 (0.9, 0.6, 0.8, -, 0.9), I 10.4 (2.9, 1.3, 3.1, 2.2, 0.9), II 9.3 (2.7, 1.2, 2.5, 2.0, 0.9), III 8.3 (2.3, 1.1, 2.1, 2.0, 0.8), IV 12.4 (3.3, 1.2, 3.1, 3.6, 1.2). Leg formula 4123. Spination of palp and legs: palp 020, 010, 010, 2012; femora I p011, d111, r021, II p011, d111, r011, III p012, d111, r112, IV p002, d111, r012; patellae I–II 000, III–IV 101; tibiae I v22212, II v22222, III–IV p11, d111, r11, v222; metatarsi I–II v222, III p112, d010, r112, v222, IV p112, r112, v2222. Chelicerae with 3 promarginal, 4 retromarginal teeth and with elongated patch of 5 tiny denticles along entire cheliceral furrow. Palpal claw with 5 secondary teeth, leg claws I–II with 5, III with 4 and IV with 7 secondary teeth. Position of tarsal organ: I 0.79, II 0.72, III 0.70, IV 1.00.

Copulatory organ (Fig. 11a and b). Lateral teeth arising posteriorly from median plate. Median plate with a large n-shaped sclerite. Copulatory openings hidden under median plate. Copulatory ducts nearly triangular. Spermathecae nearly cylindrical. Fertilisation ducts pointing anteriorly.

Colour (Fig. 11e and f). As in male.

#### Diagnosis

Small Ctenidae (total length male 5.1, female 6.4). The species resembles *A.maolan* Zhu, Chen & Song, 1999 (see Zhu et al. 1999: figs 1–5; Yin et al. 2012: fig. 473a–e; Marusik and Omelko 2016: figs 1–13 and 32–34) by having similar distal retrolateral spine, tegular apophysis (Fig. 10a–c) and fertilisation ducts (Fig. 11b), but can be distinguished by the embolus arising at 6.30 o’clock position, its tip with membranous apophysis (Fig. 10b and Fig. 14a; embolus arising centrally from tegulum, its tip without membranous apophysis in *A.maolan*), by the median plate with a large n-shaped sclerite (Fig. 11a; absent in *A.maolan*), by the lateral teeth pointing postero-medially (Fig. 11a; lateral teeth pointing medially in *A.maolan*) and by the spermathecae nearly cylindrical (Fig. 11b; spermathecae nearly wavy in *A.maolan*).

#### Etymology

The specific name refers to the type locality and is a noun in apposition.

#### Distribution

China (Tibet, type locality; Fig. 1).

#### DNA Barcode

Male (IZCAS-Ar 43536):.

TGTTTGGAGCTTGAGCTGCTATAGCTGGAACAGCAATAAGAGTTTTAATTCGAATGGAATTAGGACATTCTGGTAGATTGTTAGGAGATGATCATTTATATAATGTAATTGTAACGGCTCATGCTTTTGTTATAATTTTTTTTATAGTAATACCTATTTTGATTGGGGGCTTTGGTAATTGGTTGGTTCCTTTAATGTTAGGGGCTCCGGATATATCTTTTCCTCGAATAAATAATTTATCCTTTTGATTATTACCGCCTTCTTTATTTTTGTTGTTTATATCTTCTATAGTTGAGATAGGGGTTGGAGCAGGTTGAACGGTTTATCCTCCTTTAGCTTCTAGAATTGGGCATATGGGAAGTTCAATGGATTTTGCTATTTTTTCTTTACATTTAGCAGGTGCTTCTTCTATTATAGGTGCTGTGAATTTTATTTCTACTATTATTAATATACGATTAATAGGAATAACAATGGAGAAGATCCCTTTATTTGTATGATCGGTTTTTATTACTGCAATTTTATTATTATTATCTTTACCTGTTTTAGCAGGAGCTATTACTATATTATTGACTGATCGAAATTTTAATACTTCTTTTTTTGACCCTGCTGGAGGTGGAGATCCTATTTTATTTCAACATTTATTTTGATTTTTTG (GenBank accession number OP572101).

Female (IZCAS-Ar 43537):

TGTTTGGAGCTTGAGCTGCTATAGCTGGAACAGCAATAAGAGTTTTAATTCGAATGGAATTAGGACATTCTGGTAGATTGTTAGGAGATGATCATTTATATAATGTAATTGTAACGGCTCATGCTTTTGTTATAATTTTTTTTATAGTAATACCTATTTTGATTGGGGGCTTTGGTAATTGGTTGGTTCCTTTAATGTTAGGGGCTCCGGATATATCTTTTCCTCGAATAAATAATTTATCCTTTTGATTATTACCGCCTTCTTTATTTTTGTTGTTTATATCTTCTATAGTTGAGATAGGGGTTGGAGCAGGTTGAACGGTTTATCCTCCTTTAGCTTCTAGAATTGGGCATATGGGAAGTTCAATGGATTTTGCTATTTTTTCTTTACATTTAGCAGGTGCTTCTTCTATTATAGGTGCTGTGAATTTTATTTCTACTATTATTAATATACGATTAATAGGAATAACAATGGAGAAGATCCCTTTATTTGTATGATCGGTTTTTATTACTGCAATTTTATTATTATTATCTTTACCTGTTTTAGCAGGAGCTATTACTATATTATTGACTGATCGAAATTTTAATACTTCTTTTTTTGACCCTGCTGGAGGTGGAGATCCTATTTTATTTCAACATTTATTTTGATTTTTTG (GenBank accession number OP572102).

### Anahita popa

Jäger & Minn, 2015

7E494938-9015-535F-9FAC-80255CDB9C8C

#### Materials

- **Type status:** Other material. **Occurrence:** recordedBy: Z. Chen; individualCount: 1; sex: male; lifeStage: adult; **Taxon:** order: Araneae; family: Ctenidae; genus: Anahita; **Location:** country: Myanmar; stateProvince: Mandalay; verbatimLocality: Sagaing Hill; verbatimElevation: 168 m a.s.l.; verbatimLatitude: 21°58.595'N; verbatimLongitude: 95°59.198'E; **Event:** samplingProtocol: Collected by hand in leaf litter; year: 2017; month: 9; day: 27; **Record Level:** institutionCode: IZCAS-Ar 43538
- **Type status:** Other material. **Occurrence:** recordedBy: Z. Chen; individualCount: 1; sex: female; lifeStage: adult; **Taxon:** order: Araneae; family: Ctenidae; genus: Anahita; **Location:** country: Myanmar; stateProvince: Mandalay; verbatimLocality: Sagaing Hill; verbatimElevation: 168 m a.s.l.; verbatimLatitude: 21°58.595'N; verbatimLongitude: 95°59.198'E; **Event:** samplingProtocol: Collected by hand in leaf litter; year: 2017; month: 9; day: 27; **Record Level:** institutionCode: IZCAS-Ar 43539

#### Description

**Male** (IZCAS-Ar 43538): PL 3.3, PW 2.7, AW 0.9, OL 3.3, OW 1.8. Eye diameters and interdistances: AME 0.14, ALE 0.12, PME 0.22, PLE 0.17, AME–AME 0.08, AME–ALE 0.23, PME–PME 0.16, PME–PLE 0.21, AME–PME 0.18, ALE–PLE 0.13, clypeus AME 0.10, clypeus ALE 0.55. Palp and leg measurements: palp 3.9 (1.4, 0.6, 0.8, -, 1.1), I 15.4 (3.7, 1.6, 4.3, 3.8, 2.0), II 12.7 (3.4, 1.4, 3.3, 3.1, 1.5), III 11.0 (2.9, 1.2, 2.6, 3.1, 1.2), IV 17.3 (4.3, 1.6, 4.4, 5.2, 1.8). Leg formula 4123. Spination of palp and legs: palp 151, 000, 122; femora I p021, d111, r112, II p112, d111, r112, III p111, d111, r111, IV p012, d111, r112; patellae I–IV 101; tibiae I p010, d101, r110, v22222, II p10, d101, r110, v22222, III–IV p11, d111, r11, v222; metatarsi I p111, d001, r111, v222, II p111, d111, r111, v222, III p111, d012, r111, v222, IV p112, d011, r112, v222. Chelicerae with 3 promarginal, 4 + 2 retromarginal teeth and with elongated patch of 3 tiny denticles along entire cheliceral furrow. Retromargin of chelicerae close to fang base with 2 bristles. Sparse scopula restricted almost entirely to tarsi. Leg claws I with 6, II–III with 5 and IV with 4secondary teeth. Position of tarsal organ: I 1.89, II 1.03, III 0.75, IV 1.56.

**Figure 12.**
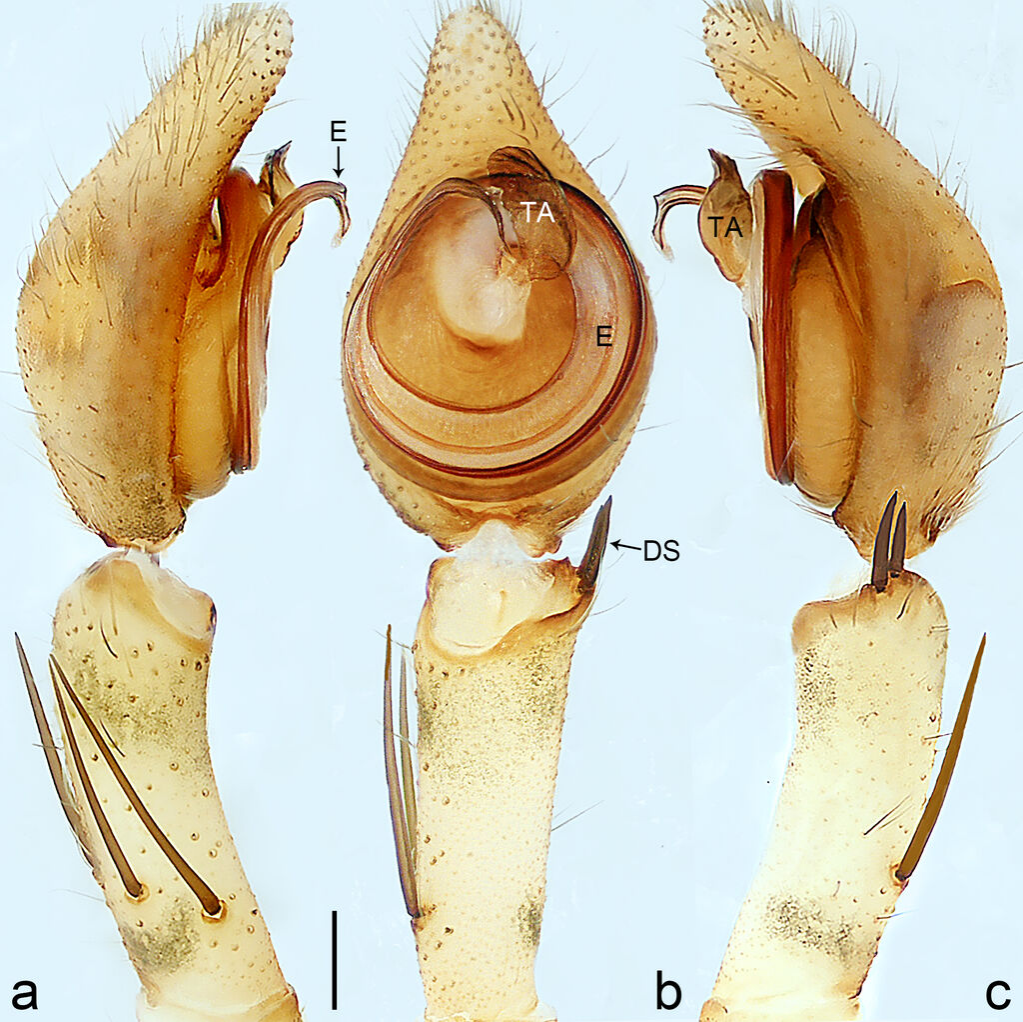
*Anahitapopa* Jäger & Minn, 2015, palp, male from Sagaing Hill. **a**: Prolateral view; **b**: Ventral view; **c**: Retrolateral view. DS = distal retrolateral spine, E = embolus, TA = tegular apophysis. Scale bar: 0.2 mm (**a–c**).

Palp (Fig. 12a–c). Palpal tibia without RTA and intrasegmental sclerite, distally with 2 stout retrolateral spines. Cymbium tip slightly conical. Embolus (Fig. 14b) arising at 12 o’clock position, long and laminar, running around tegulum, its tip situated distally. Conductor absent. Tegular apophysis arising at 12 to 12.30 o’clock position subdistally.

**Figure 13.**
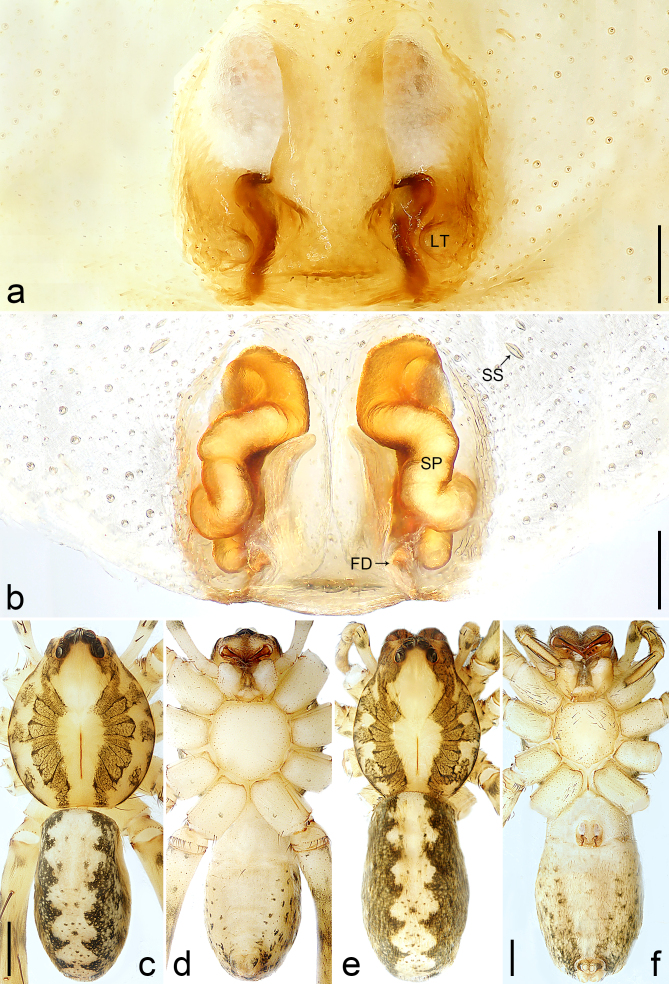
*Anahitapopa* Jäger & Minn, 2015, male and female from Sagaing Hill. **a**: Female, epigyne, ventral view; **b**: Same, vulva, dorsal view; **c**: Male, habitus, dorsal view; **d**: Same, habitus, ventral view; **e**: Female, habitus, dorsal view; **f**: Same, habitus, ventral view. FD = fertilisation duct, LT = lateral teeth, SP = spermathecae, SS = slit sensillum. Scale bars: 0.2 mm (**a**, **b**), 1.0 mm (**c–f**).

Colour (Fig. 13c and d). Yellowish-brown with light brown markings. Dorsal prosoma with distinct light median band and dark lateral bands, light patches partly fused, frontally with 2 light patches close to ALE. Sternum, coxae, labium and gnathocoxae pale yellowish without patterns. Chelicerae yellowish-brown with longitudinal dark lines frontally. Palps and legs yellowish-brown, legs I–III with pattern especially from femora to tibiae. Dorsal opisthosoma yellowish-brown with distinct serrated light median band. Lateral opisthosoma spotted. Ventral opisthosoma yellowish, with posteriorly converging lines of spots. Spinnerets light, anterior lateral spinnerets laterally dark, anal tubercle light.

**Female** (IZCAS-Ar 43539): See Fig. 13a, b, e and f; figs 1–6 in Jäger and Minn (2015).

#### Diagnosis

Small Ctenidae (total length male 6.6). The species can be distinguished from all known congeners by the embolus arising at 12 o’clock position, long and laminar, running around tegulum, its tip situated distally (Fig. 12b and Fig. 14b), by the palp having no conductor (Fig. 12a–c), by the tegular apophysis arising at 12 to 12.30 o’clock position subdistally (Fig. 12b) and by the tibia distally with 2 stout retrolateral spines (Fig. 12b and c). For the diagnosis of female, see Jäger and Minn (2015).

#### Distribution

Myanmar (Sagaing Hill, Fig. 1; Mt Popa, type locality).

#### DNA Barcode

Male (IZCAS-Ar 43538):

TATTTGGGGCTTGAGCTGCTATAGCGGGTACTGCAATAAGAGTTTTGATTCGAATGGAATTAGGACATCCTGGAAGATTATTAGGTGATGATCATTTATATAATGTTATTGTAACAGCTCATGCTTTTGTTATGATTTTTTTTATAGTTATACCTATTTTAATTGGTGGTTTTGGAAATTGGTTAGTTCCTTTAATATTAGGAGCTCCGGATATATCATTTCCTCGAATAAATAATTTATCTTTTTGGTTATTACCTCCTTCTTTGTTTTTATTGTTTATATCTTCTATAGTTGAAATAGGTGTAGGAGCAGGGTGAACAGTTTATCCTCCTTTAGCTTCTAGAATTGGGCATGCAGGGAGATCTATGGATTTTGCTATTTTTTCTTTACATTTAGCGGGTGCTTCTTCTATTATAGGGGCTGTAAATTTTATTTCTACTATTATTAATATACGATTAATAGGAATGACTATAGAGAAGGTTCCTTTGTTTGTTTGATCTGTTTTTATTACTGCAATTTTATTATTGTTATCTTTACCAGTGTTAGCTGGTGCTATTACAATATTATTAACTGATCGTAATTTTAATACTTCTTTTTTTGATCCTGCTGGAGGAGGAGATCCAGTTTTATTTCAGCATTTGTTTTGATTTTTTG (GenBank accession number OP572105).

Female (IZCAS-Ar 43539):

TTTTTGGAGCTTGAGCCGCTATAGCGGGTACTGCAATAAGAGTTTTAATTCGAATAGAATTAGGGCATCCTGGGAGATTATTAGGTGATGATCATTTATATAATGTTATTGTAACAGCTCATGCTTTTGTTATAATTTTTTTTATAGTTATACCTATTTTAATTGGTGGTTTTGGAAATTGGTTAGTTCCTTTAATGTTAGGAGCTCCGGATATATCATTTCCTCGAATAAATAATTTATCTTTTTGATTATTACCTCCTTCTTTGTTTTTATTGTTTATATCTTCCATGGTTGAAATAGGTGTGGGAGCAGGATGGACAGTTTATCCTCCTTTAGCTTCTAGAATTGGGCATGCGGGAAGATCTATGGATTTTGCTATTTTTTCTTTACATTTAGCGGGTGCTTCTTCTATTATAGGAGCTGTAAATTTTATTTCGACTATTATTAATATACGATTAATAGGAATGACTATAGAGAAGGTTCCCTTATTTGTTTGATCTGTTTTTATTACTGCAATTTTATTGTTATTATCTTTACCAGTATTAGCTGGTGCTATTACGATGTTGTTAACTGATCGTAATTTTAATACTTCTTTTTTTGACCCTGCTGGGGGAGGGGATCCGGTTTTATTTCAACATTTATTTTGATTTTTTG (GenBank accession number OP572104).

### Bowie fascination

Jäger, 2022

17AE53CC-49AB-5805-BCFB-4FD7C159B895

#### Materials

- **Type status:** Other material. **Occurrence:** recordedBy: F. Gao; individualCount: 1; sex: male; lifeStage: adult; **Taxon:** order: Araneae; family: Ctenidae; genus: Bowie; **Location:** country: China; stateProvince: Yunnan; municipality: Puer; locality: Jiangcheng County; verbatimLatitude: 22°35.640'N; verbatimLongitude: 101°50.760'E; **Event:** samplingProtocol: Collected by hand in leaf litter; year: 2022; month: 7; day: 19–24; **Record Level:** institutionCode: IZCAS-Ar 43540
- **Type status:** Other material. **Occurrence:** recordedBy: F. Gao; individualCount: 1; sex: female; lifeStage: adult; **Taxon:** order: Araneae; family: Ctenidae; genus: Bowie; **Location:** country: China; stateProvince: Yunnan; municipality: Puer; locality: Jiangcheng County; verbatimLatitude: 22°35.640'N; verbatimLongitude: 101°50.760'E; **Event:** samplingProtocol: Collected by hand in leaf litter; year: 2022; month: 7; day: 19–24; **Record Level:** institutionCode: IZCAS-Ar 43541

#### Description

**Male** (IZCAS-Ar 43540): See Fig. 15a–c, Fig. 16c, d and Fig. 28a; figs 230–233 and 263–264 in Jäger (2022).

**Female** (IZCAS-Ar 43541): PL 9.9, PW 7.8, AW 4.1, OL 11.0, OW 7.3. Eye diameters and interdistances: AME 0.29, ALE 0.27, PME 0.32, PLE 0.39, AME–AME 0.31, AME–ALE 0.71, PME–PME 0.43, PME–PLE 1.32, AME–PME 0.32, ALE–PLE 0.38, clypeus AME 0.42, clypeus ALE 0.95. Palp and leg measurements: palp 10.0 (3.3, 1.9, 2.1, -, 2.7), I 23.0 (6.7, 3.9, 5.7, 5.0, 1.7), II 21.4 (6.4, 3.7, 5.1, 4.6, 1.6), III 18.6 (5.6, 3.0, 3.8, 4.6, 1.6), IV 26.1 (7.2, 3.4, 5.8, 7.6, 2.1). Leg formula 4123. Spination of palp and legs: palp 131, 100, 131, 3020; femora I p021, d111, r111, II p112, d111, r111, III–IV p111, d111, r112; patellae I–II 000, III–IV 101; tibiae I–II v22222, III–IV p11, d111, r11, v222; metatarsi I–II v222, III p111, d012, r111, v222, IV p121, d012, r111, v2122. Chelicerae with 3 promarginal, 4 + 1 retromarginal teeth and with elongated patch of 27 tiny denticles along entire cheliceral furrow. Retromargin of chelicerae close to fang base with 11 bristles. Ventral tarsi and metatarsi I–II with sparse scopula. Palpal claw with 5 secondary teeth, leg claws I–II with 1, III with 2 and IV with 3 secondary teeth. Position of tarsal organ: I 1.41, II 1.34, III 1.25, IV 1.44.

**Figure 16.**
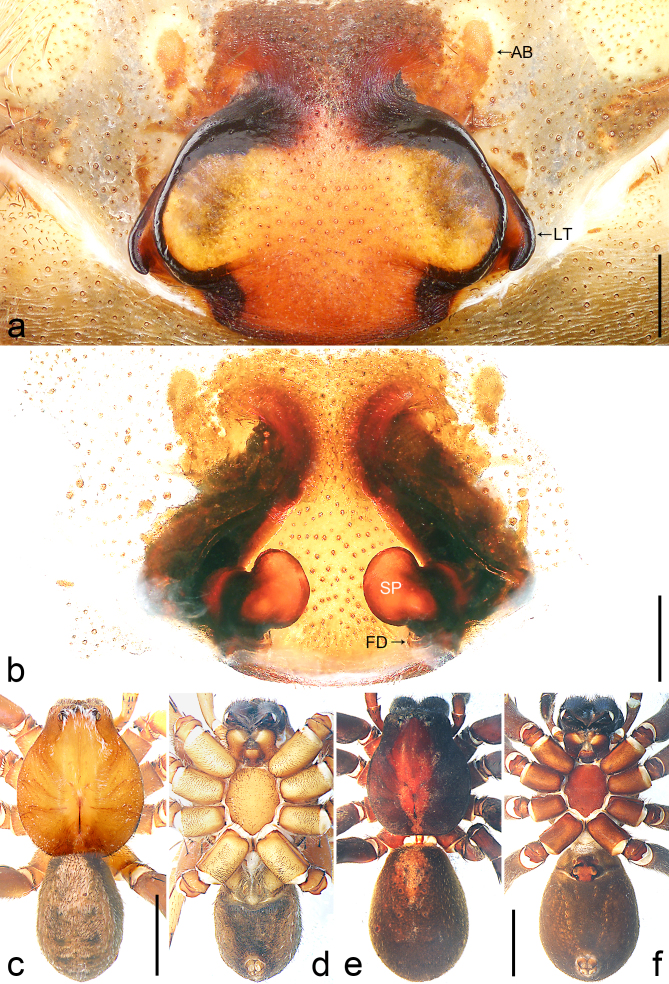
*Bowiefascination* Jäger, 2022, male and female from Yunnan. **a**: Female, epigyne, ventral view; **b**: Same, vulva, dorsal view; **c**: Male, habitus, dorsal view; **d**: Same, habitus, ventral view; **e**: Female, habitus, dorsal view; **f**: Same, habitus, ventral view. AB = anterior band of epigynal field, FD = fertilisation duct, LT = lateral teeth, SP = spermathecae. Scale bars: 0.5 mm (**a**, **b**), 5.0 mm (**c–f**).

Copulatory organ (Fig. 16a, b and Fig. 21a). Epigynal field wider than long, anterior bands separated from epigynal field. Lateral teeth arising laterally from median plate, curved and with pointed tips. Median plate with constricted posterior part and without distinct humped areas in posterior view. Internal duct system with two large vulval folds. Spermathecae separated by less than their diameter, with distinctly developed two chambers, smaller chamber with distinct external rim. Fertilisation ducts pointing laterally.

Colour (Fig. 16e and f). Deep reddish-brown with darker patterns. Dorsal prosoma with characteristic lighter median band, widened behind eyes, eye field with sparse white hairs and with distinctly marked fovea and radial markings. Sternum, labium, gnathocoxae and ventral coxae reddish-brown without patterns and gnathocoxae with lighter distal lips. Chelicerae black. Palps and legs deep reddish-brown. Dorsal opisthosoma brown with black patches, anterior margin with lighter area. Lateral opisthosoma spotted. Ventral opisthosoma brown with posteriorly converging lines of spots. Anterior lateral spinnerets laterally dark, posterior lateral and median spinnerets and anal tubercle light.

#### Diagnosis

Large-sized Ctenidae (total length female 20.9). The species is assigned to the *robustus*-species group with the characteristics of stout tegular apophysis, simple stout embolus with broad base, presence of retro-proximal cymbial outgrowth, RTA arising medially to distally from palpal tibia, female possesses a transversally median plate with lateral teeth situated mainly laterally and not reaching the epigastric furrow. It resembles *B.candidate* Jäger, 2022 (see Jäger 2022: figs 254–262 and 280–284) by having similar lateral teeth (Fig. 16a and Fig. 21a), but can be distinguished by the median plate without distinct humped areas (Fig. 21a; median plate with humped areas best seen in posterior view in *B.candidate*), by the spermathecae separated by less than their diameter and smaller chamber with distinct external rim (Fig. 16b, spermathecae separated by about their diameter and smaller chamber without distinct external rim in *B.candidate*) and by the fertilisation ducts pointing laterally (Fig. 16b, fertilisation ducts pointing antero-medially in *B.candidate*). For the diagnosis of male, see Jäger (2022).

#### Distribution

China (Yunnan, Fig. 1); Vietnam (Dien Bien, type locality).

#### DNA Barcode

Male (IZCAS-Ar 43540):

TATTTGGATCTTGGGCTGCTATAGCTGGGACAGCTATAAGAGTATTAATTCGTATAGAGCTAGGTCATTCTGGTAGATTATTTGGTGATGATCATTTATATAATGTAATTGTTACAGCTCATGCTTTTGTAATAATTTTTTTTATGGTTATGCCTATTTTAATTGGTGGTTTTGGAAACTGATTAGTTCCTTTGATATTAGGGGCTCCTGATATATCTTTTCCTCGTATAAATAATTTATCTTTTTGATTACTCCCTCCTTCATTATTTTTGTTATTTATATCTTCTATGGTTGAGATAGGGGTGGGAGCTGGTTGGACAGTGTATCCTCCTTTAGCTTCTAGTATTGGCCATATAGGAAGATCAATAGATTTTGCTATTTTTTCTTTACATTTAGCGGGAGCTTCTTCTATTATAGGGGCTGTTAATTTTATTTCTACAATTATTAATATACGTTTGTATGGAGTAAGAATAGAAAAGGTGCCTTTATTTGTATGATCTGTTCTAATTACTGCAGTATTATTGCTTTTATCTTTACCTGTATTAGCAGGTGCTATTACTATATTATTAACTGATCGTAATTTTAATACTTCTTTTTTTGACCCGGCTGGAGGAGGGGATCCAGTTTTATTTCAACATTTATTTTGATTTTTTG (GenBank accession number OP572108).

Female (IZCAS-Ar 43541):

TATTTGGATCTTGGGCTGCTATAGCTGGGACAGCTATAAGAGTATTAATTCGTATAGAGCTAGGTCATTCTGGTAGATTATTTGGTGATGATCATTTATATAATGTAATTGTTACAGCTCATGCTTTTGTAATAATTTTTTTTATGGTTATGCCTATTTTAATTGGTGGTTTTGGAAACTGATTAGTTCCTTTGATATTAGGGGCTCCTGATATATCTTTTCCTCGTATAAATAATTTATCTTTTTGATTACTCCCTCCTTCATTATTTTTGTTATTTATATCTTCTATGGTTGAGATAGGGGTGGGAGCTGGTTGGACAGTGTATCCTCCTTTAGCTTCTAGTATTGGCCATATAGGAAGATCAATAGATTTTGCTATTTTTTCTTTACATTTAGCGGGAGCTTCTTCTATTATAGGGGCTGTTAATTTTATTTCTACAATTATTAATATACGTTTGTATGGAGTAAGAATAGAAAAGGTGCCTTTATTTGTATGATCTGTTCTAATTACTGCAGTATTATTGCTTTTATCTTTACCTGTATTAGCAGGTGCTATTACTATATTATTAACTGATCGTAATTTTAATACTTCTTTTTTTGACCCGGCTGGAGGAGGGGATCCAGTTTTATTTCAACATTTATTTTGATTTTTTG (GenBank accession number OP572107).

### Bowie ninhbinh

S. Li & Yao sp. n.

830F2587-709C-5BBB-B737-FDE9E22C3211

A347DE57-4669-435E-8A34-D2139FFAFDBF

#### Materials

- **Type status:** Holotype. **Occurrence:** recordedBy: Z. Chen; individualCount: 1; sex: male; lifeStage: adult; **Taxon:** order: Araneae; family: Ctenidae; genus: Bowie; **Location:** country: Vietnam; stateProvince: Ninh Binh; verbatimLocality: Cuc Phuong National Park; verbatimElevation: 158 m a.s.l.; verbatimLatitude: 20°15.006'N; verbatimLongitude: 105°42.895'E; **Event:** samplingProtocol: Collected by hand in leaf litter; year: 2015; month: 8; day: 19; **Record Level:** institutionCode: IZCAS-Ar 43542
- **Type status:** Paratype. **Occurrence:** recordedBy: Z. Chen; individualCount: 1; sex: male; lifeStage: adult; **Taxon:** order: Araneae; family: Ctenidae; genus: Bowie; **Location:** country: Vietnam; stateProvince: Ninh Binh; verbatimLocality: Cuc Phuong National Park; verbatimElevation: 158 m a.s.l.; verbatimLatitude: 20°15.006'N; verbatimLongitude: 105°42.895'E; **Event:** samplingProtocol: Collected by hand in leaf litter; year: 2015; month: 8; day: 20; **Record Level:** institutionCode: IZCAS-Ar 43543

#### Description

**Male** (IZCAS-Ar 43542): PL 7.6, PW 5.9, AW 3.0, OL 5.8, OW 3.9. Eye diameters and interdistances: AME 0.26, ALE 0.19, PME 0.38, PLE 0.30, AME–AME 0.19, AME–ALE 0.29, PME–PME 0.22, PME–PLE 0.43, AME–PME 0.18, ALE–PLE 0.23, clypeus AME 0.11, clypeus ALE 0.55. Palp and leg measurements: palp 7.8 (2.7, 1.2, 1.3, -, 2.6), I 21.3 (5.6, 3.0, 5.8, 4.9, 2.0), II 19.6 (5.3, 2.7, 5.1, 4.7, 1.8), III 15.9 (4.3, 2.5, 3.4, 4.1, 1.6), IV 23.1 (5.9, 2.5, 5.7, 7.0, 2.0). Leg formula 4123. Spination of palp and legs: palp 151, 100, 101; femora I p021, d111, r112, II p112, d111, r112, III p212, d111, r112, IV p112, d111, r012; patellae I–IV 101; tibiae I p110, d111, r210, v22222, II p110, d111, r110, v22222, III p11, d200, r11, v222, IV p11, d111, r11, v222; metatarsi I–III p112, d010, r112, v222, IV p112, d010, r112, v2222. Chelicerae with 3 promarginal, 4 retromarginal teeth and with elongated patch of 19 tiny denticles along entire cheliceral furrow. Retromargin of chelicerae close to fang base with 6 bristles. Ventral tarsi and metatarsi I–II with sparse scopula. Right leg claws I–III with 2 and IV with 3 secondary teeth. Position of tarsal organ: I 1.27, II 1.28, III 1.06, IV 1.36.

**Figure 17.**
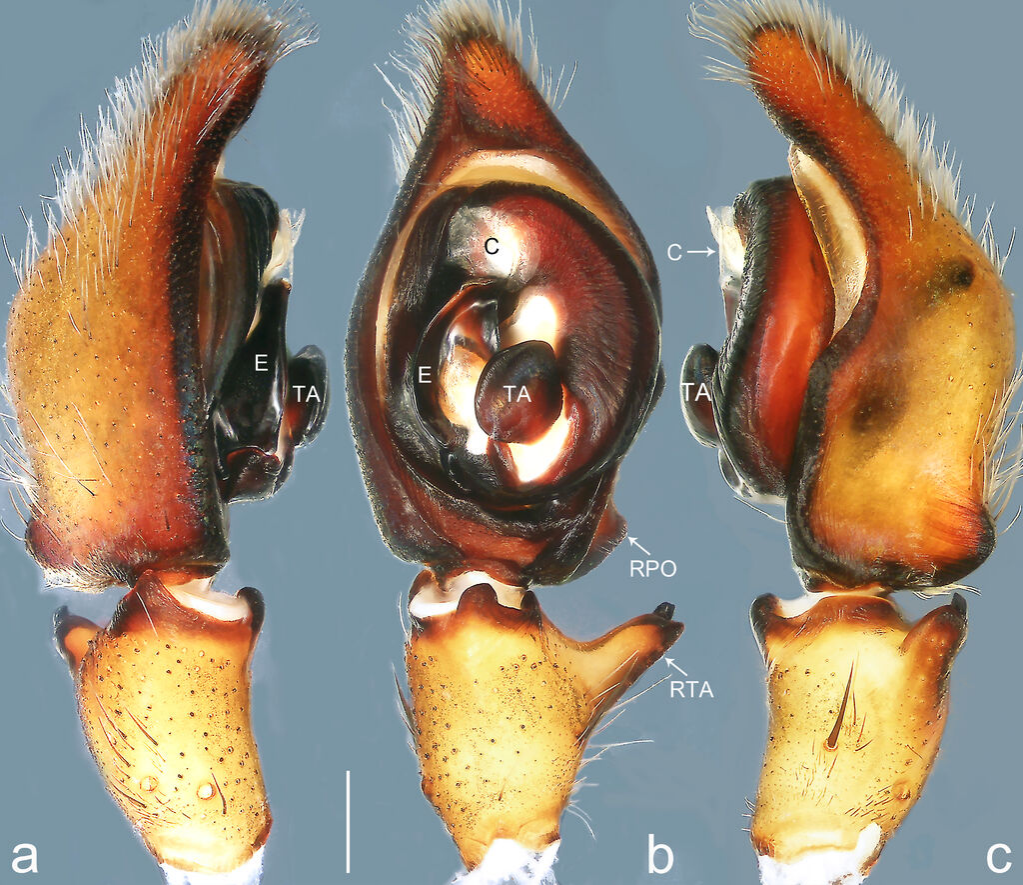
*Bowieninhbinh* sp. n., palp, holotype. **a**: Prolateral view; **b**: Ventral view; **c**: Retrolateral view. C = conductor, E = embolus, RPO = retro-proximal cymbial outgrowth, RTA = retrolateral tibial apophysis, TA = tegular apophysis. Scale bar: 0.5 mm (**a–c**).

Palp (Fig. 17a–c). RTA arising from tibia subdistally, stout and distally bifurcated. Cymbium tip slightly conical, with retro-proximally outgrowth. Embolus (Fig. 28b) arising at 7.30 o’clock position, its tip wide and blunt, situated in distal half of tegulum. Conductor arising at 12 o’clock position. Tegular apophysis arising subcentrally from tegulum, nearly round.

**Figure 18.**
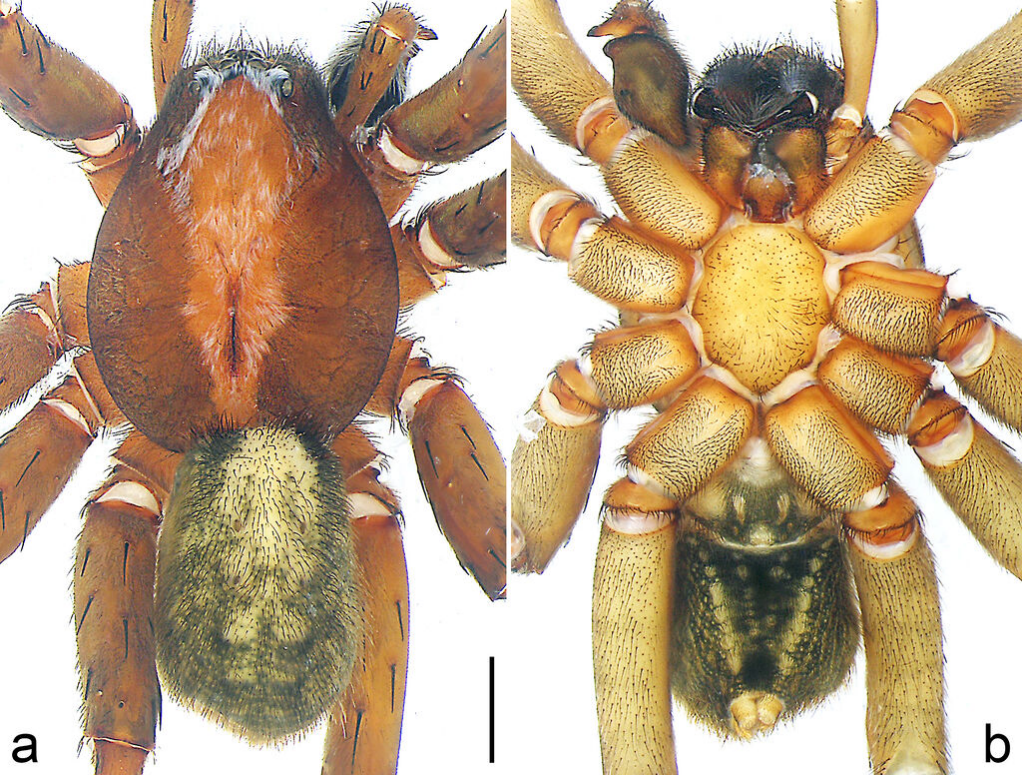
*Bowieninhbinh* sp. n., habitus, holotype male. **a**: Dorsal view; **b**: Ventral view. Scale bar: 2.0 mm (**a**, **b**).

Colour (Fig. 18a and b). Reddish-brown to yellowish with dark patterns. Dorsal prosoma with characteristic lighter median band, widened behind eyes and with some white hairs, distinctly marked fovea and indistinct radial markings. Sternum and ventral coxae yellowish, labium brown and gnathocoxae brown with dark patterns. Chelicerae reddish-brown with longitudinal lines. Legs reddish brown-yellowish. Dorsal opisthosoma yellowish with black patches. Lateral opisthosoma yellowish with darker spots. Ventral opisthosoma yellowish with dark patterns; epiandrium and muscle sigilla light. Spinnerets and anal tubercle light.

**Female**

Unknown.

**Variation**: Paratype male (IZCAS-Ar 43543): PL 7.4, OL 5.7.

#### Diagnosis

Medium-sized Ctenidae (total length male 13.1–13.4). The new species is assigned to the *robustus*-species group with the characteristics of stout tegular apophysis, simple stout embolus with broad base, presence of retro-proximal cymbial outgrowth and RTA arising subdistally from palpal tibia. It resembles *B.dodo* Jäger, 2022 (see Jäger 2022: figs 241–243 and 267–268) by having similar conductor, broad embolus and retro-proximal cymbial outgrowth (Fig. 17a–c and Fig. 28b), but can be distinguished by the tegular apophysis nearly round and without concave (Fig. 17b, tegular apophysis with distinct concave on retrolateral side in *B.dodo*) and by the RTA distally bifurcated (Fig. 17b, RTA having no bifurcation in *B.dodo*).

#### Etymology

The specific name refers to the type locality and is a noun in apposition.

#### Distribution

Vietnam (Ninh Binh, type locality; Fig. 1).

#### DNA Barcode

Male (IZCAS-Ar 43542):

TACTTGGATCTTGGGCTGCTATGGCAGGGACAGCTATAAGAGTATTAATTCGGATGGAATTAGGCCATTCTGGGAGATTGTTAGGTGATGATCATTTATACAATGTAATTGTTACTGCACATGCTTTTGTAATGATTTTTTTTATAGTAATGCCTATTTTAATTGGGGGTTTTGGAAATTGGTTAGTACCTTTGATATTAGGGGCTCCTGATATATCTTTTCCTCGAATAAATAATTTGTCTTTTTGGTTACTTCCTCCTTCGTTATTTTTATTATTTATATCTTCAATAGTTGAGATAGGAGTTGGAGCTGGATGAACGGTATATCCTCCTTTAGCTTCTAGTATTGGTCATATAGGGAGATCTATAGATTTTGCTATTTTTTCTTTACATTTAGCGGGGGCTTCTTCTATTATAGGAGCGGTAAATTTTATTTCTACGATTATTAATATGCGTTTGTATGGGATGACTATAGAGAAAGTACCTTTATTTGTGTGATCTGTTTTAATTACTGCGGTATTGTTATTATTGTCTTTACCTGTTTTAGCAGGTGCTATTACTATATTGTTAACTGATCGAAATTTTAATACTTCTTTTTTTGATCCGGCTGGGGGTGGTGATCCTGTTTTGTTTCAACATTTATTTTGATTTTTTG (GenBank accession number OP572110).

### Bowie vinhphuc

S. Li & Yao sp. n.

729CAD34-216B-57FB-97AC-D01B41FAE4D1

B093C735-BBC5-41AB-9880-FAFD5B3D5BB6

#### Materials

- **Type status:** Holotype. **Occurrence:** recordedBy: D. Pham; individualCount: 1; sex: male; lifeStage: adult; **Taxon:** order: Araneae; family: Ctenidae; genus: Bowie; **Location:** country: Vietnam; stateProvince: Vinh Phuc; verbatimLocality: Tam Dao National Park; verbatimElevation: 172 m a.s.l.; verbatimLatitude: 21°22.733'N; verbatimLongitude: 105°32.817'E; **Event:** samplingProtocol: Collected by hand in leaf litter; year: 2003; month: 5; day: 6; **Record Level:** institutionCode: IZCAS-Ar 43544
- **Type status:** Paratype. **Occurrence:** recordedBy: D. Pham; individualCount: 1; sex: male; lifeStage: adult; **Taxon:** order: Araneae; family: Ctenidae; genus: Bowie; **Location:** country: Vietnam; stateProvince: Vinh Phuc; verbatimLocality: Tam Dao National Park; verbatimElevation: 172 m a.s.l.; verbatimLatitude: 21°22.733'N; verbatimLongitude: 105°32.817'E; **Event:** samplingProtocol: Collected by hand in leaf litter; year: 2003; month: 5; day: 6; **Record Level:** institutionCode: IZCAS-Ar 43545
- **Type status:** Paratype. **Occurrence:** recordedBy: D. Pham; individualCount: 1; sex: female; lifeStage: adult; **Taxon:** order: Araneae; family: Ctenidae; genus: Bowie; **Location:** country: Vietnam; stateProvince: Vinh Phuc; verbatimLocality: Tam Dao National Park; verbatimElevation: 172 m a.s.l.; verbatimLatitude: 21°22.733'N; verbatimLongitude: 105°32.817'E; **Event:** samplingProtocol: Collected by hand in leaf litter; year: 2003; month: 5; day: 6; **Record Level:** institutionCode: IZCAS-Ar 43728
- **Type status:** Paratype. **Occurrence:** recordedBy: D. Pham; individualCount: 1; sex: female; lifeStage: adult; **Taxon:** order: Araneae; family: Ctenidae; genus: Bowie; **Location:** country: Vietnam; stateProvince: Vinh Phuc; verbatimLocality: Tam Dao National Park; verbatimElevation: 172 m a.s.l.; verbatimLatitude: 21°22.733'N; verbatimLongitude: 105°32.817'E; **Event:** samplingProtocol: Collected by hand in leaf litter; year: 2003; month: 5; day: 6; **Record Level:** institutionCode: IZCAS-Ar 43729

#### Description

**Male** (IZCAS-Ar 43544): PL 6.4, PW 5.0, AW 2.4, OL 3.1, OW 3.5. Eye diameters and interdistances: AME 0.23, ALE 0.24, PME 0.28, PLE 0.26, AME–AME 0.22, AME–ALE 0.42, PME–PME 0.25, PME–PLE 0.41, AME–PME 0.20, ALE–PLE 0.21, clypeus AME 0.21, clypeus ALE 0.53. Palp and leg measurements: palp 7.4 (2.8, 0.9, 1.4, -, 2.3), I 17.7 (5.2, 2.5, 4.3, 4.3, 1.4), II 15.9 (4.7, 2.4, 3.7, 3.8, 1.3), III 13.4 (3.8, 1.9, 2.9, 3.6, 1.2), IV 19.5 (5.5, 2.3, 4.3, 5.8, 1.6). Leg formula 4123. Spination of palp and legs: palp 141, 100, 1010; femora I p021, d111, r112, II p112, d111, r1111, III p1111, d111, r1111, IV p002, d111, r111; patellae I–IV 101; tibiae I p110, d111, r21, v22222, II p20, d111, r110, v22222, III–IV p11, d111, r11, v222; metatarsi I p11, d002, r111, v222, II p111, r111, v222, III p111, d012, r211, v222, IV p122, d012, r111, v2122. Chelicerae with 3 promarginal, 4 retromarginal teeth and with elongated patch of 12 tiny denticles along entire cheliceral furrow. Retromargin of chelicerae close to fang base with 4 bristles. Sparse scopula restricted almost entirely to tarsi, only metatarsi I–II with sparse scopula hairs. Leg claws I–III with 3 and IV with 4 secondary teeth. Position of tarsal organ: I with 1.17, II 1.07, III 0.90, IV 1.09.

**Figure 19.**
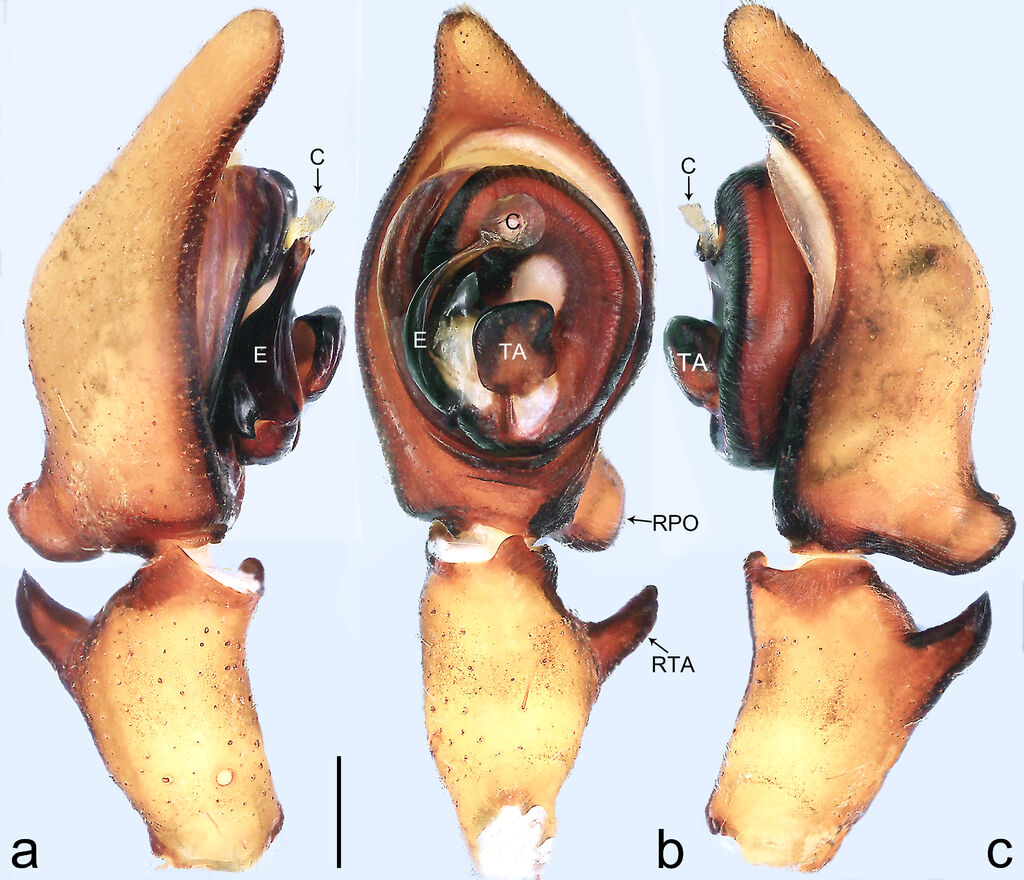
*Bowievinhphuc* sp. n., palp, holotype. **a**: Prolateral view; **b**: Ventral view; **c**: Retrolateral view. C = conductor, E = embolus, RPO = retro-proximal cymbial outgrowth, RTA = retrolateral tibial apophysis, TA = tegular apophysis. Scale bar: 0.5 mm (**a–c**).

Palp (Fig. 19a–c). RTA arising from tibia subdistally, with wide base and narrow tip. Cymbium tip slightly conical and with retrolatero-proximal outgrowth. Embolus (Fig. 28c) arising at 7 o’clock position, with a large seam from the middle to the tip. Conductor arising at 12 o’clock position. Tegular apophysis arising at 6 o’clock position, covering partly membranous extension part of embolus.

**Figure 20.**
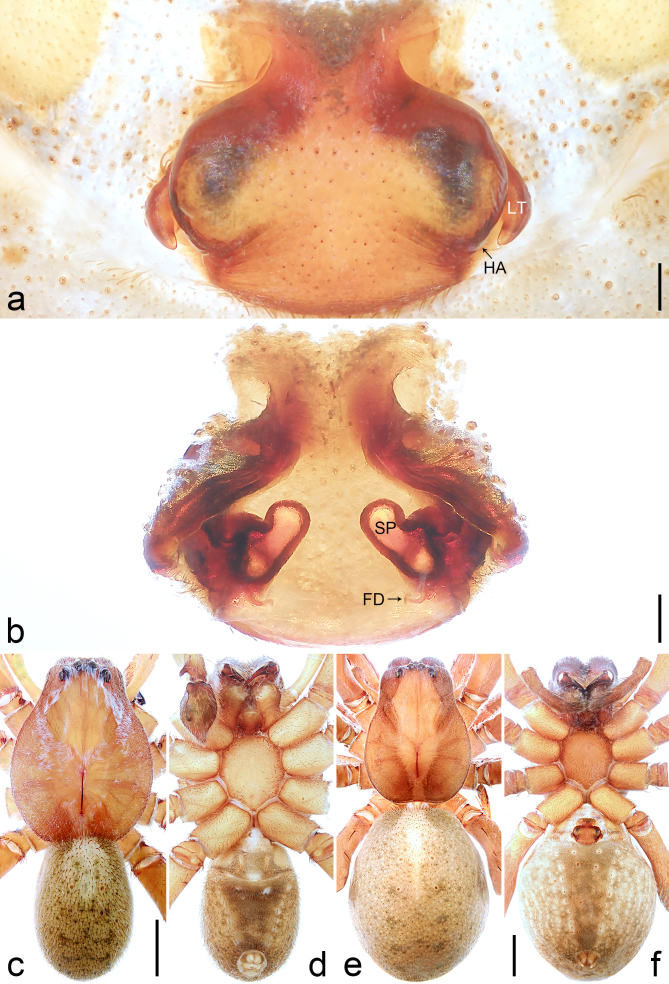
*Bowievinhphuc* sp. n. **a**: Paratype female, epigyne, ventral view; **b**: Same, vulva, dorsal view; **c**: Holotype male, habitus, dorsal view; **d**: Same, habitus, ventral view; **e**: Paratype female, habitus, dorsal view; **f**: Same, habitus, ventral view. FD = fertilisation duct, HA = humped areas of median plate, LT = lateral teeth, SP = spermathecae. Scale bars: 0.2 mm (**a**, **b**), 2.0 mm (**c–f**).

Colour (Fig. 20c and d). Deep reddish-brown with darker patterns. Dorsal prosoma with characteristic lighter median band, widened behind eyes, eye field and lateral field with white dense hairs, with distinctly marked fovea and radial markings. Sternum and ventral coxae yellowish-brown without patches, labium and gnathocoxae reddish-brown with lighter distal lips. Chelicerae deep reddish-brown without patterns. Palps and legs reddish-brown without patterns. Dorsal opisthosoma yellowish-brown with black patches and anterior margin with lighter area. Lateral opisthosoma without spots. Ventral opisthosoma dark brown with posteriorly converging lines of spots. Anterior lateral spinnerets laterally dark, posterior lateral and median spinnerets and anal tubercle light.

**Female** (IZCAS-Ar 43728): PL 6.7, PW 5.2, AW 3.1, OL 8.5, OW 7.0. Eye diameters and interdistances: AME 0.25, ALE 0.21, PME 0.28, PLE 0.26, AME–AME 0.22, AME–ALE 0.48, PME–PME 0.29, PME–PLE 0.57, AME–PME 0.23, ALE–PLE 0.23, clypeus AME 0.19, clypeus ALE 0.58. Palp and leg measurements: palp 6.9 (2.5, 1.3, 1.4, -, 1.7), I 16.3 (4.8, 2.6, 4.1, 3.5, 1.3), II 15.0 (4.5, 2.4, 3.5, 3.4, 1.2), III 13.1 (4.1, 2.0, 2.7, 3.1, 1.2), IV 18.8 (5.2, 2.2, 4.1, 5.6, 1.7). Leg formula 4123. Spination of palp and legs: palp 131, 000, 1111, 2101; femora I p021, d111, r021, II p112, d111, r021, III p112, d111, r112, IV p111, d111, r001; patellae I–II 000, III–IV 101, tibiae I–II v22222, III–IV p11, d111, r11, v222; metatarsi I–II v222, III p112, d010, r112, v222, IV p112, r112, v2222. Chelicerae with 3 promarginal, 4 retromarginal teeth and with elongated patch of 10 tiny denticles along entire cheliceral furrow. Retromargin of chelicerae close to fang base with 5 bristles. Sparse scopula restricted almost entirely to tarsi, only metatarsi I–II with sparse scopula hairs. Palpal claw with 6 secondary teeth, leg claws I with 1, II–III with 2 and IV with 3 secondary teeth. Position of tarsal organ: I 1.03, II 0.97, III 0.95, IV 1.42.

**Figure 21.**
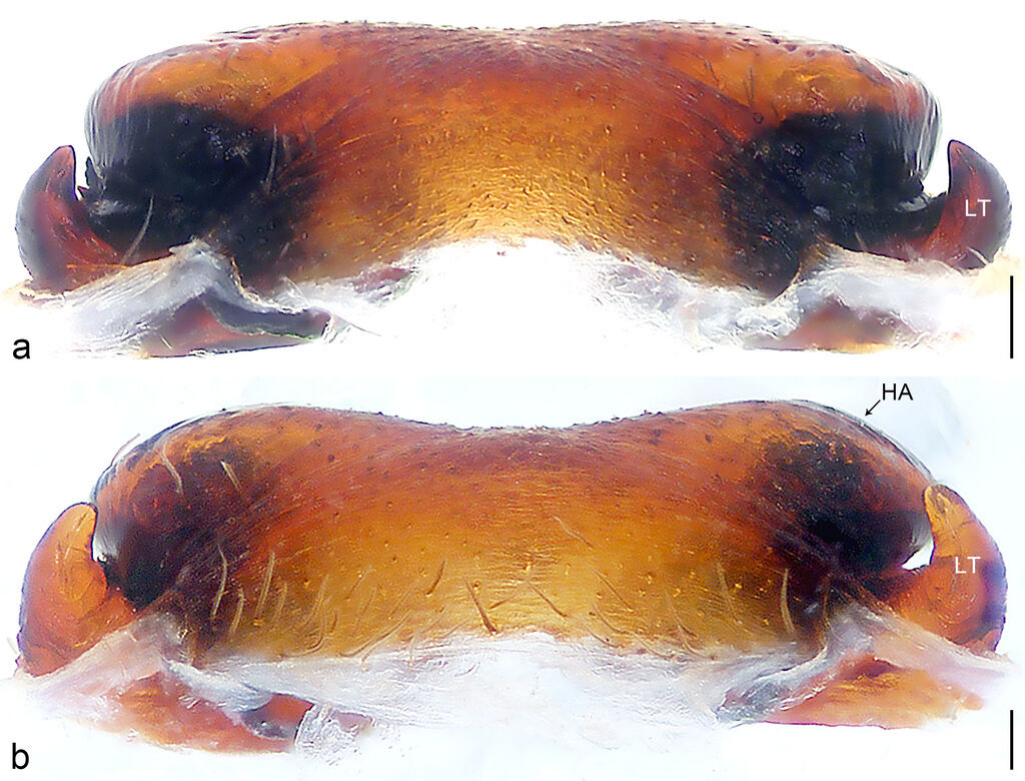
Epigyne, posterior view. **a**: *Bowiefascination* Jäger, 2022; **b**: *B.vinhphuc* sp. n. HA = humped areas of median plate, LT = lateral teeth. Scale bars: 0.2 mm (**a**), 0.1 mm (**b**).

Copulatory organ (Fig. 20a, b and Fig. 21b). Epigynal field wider than long. Lateral teeth arising laterally from median plate, curved and with pointed tips. Median plate with constricted posterior part and distinct humped areas best seen in posterior view. Internal duct system with two large vulval folds. Spermathecae separated by less than their diameter, larger chamber round and apically swollen and smaller one spherical. Fertilisation ducts pointing medially.

Colour (Fig. 20e and f). As in male, except for: dorsal prosoma with distinct light median band and without white dense hairs. Lateral opisthosoma spotted.

**Variation**: Paratype male (IZCAS-Ar 43545): PL 7.4, OL 6.5. Second paratype female (IZCAS-Ar 43729): PL 7.6, OL 7.8.

#### Diagnosis

Small to medium-sized Ctenidae (total length male 9.5–13.9, female 15.2–15.4). The new species is assigned to the *robustus*-species group with the characteristics of stout tegular apophysis, simple stout embolus with broad base and short apical part, presence of retro-proximal cymbial outgrowth, RTA arising subdistally from palpal tibia, female possesses a transversally oval median plate with lateral teeth situated laterally and not reaching the epigastric furrow. It resembles *B.yassassin* Jäger, 2022 (see Jäger 2022: figs 287–290 and 307–313) by having similar retro-proximal cymbial outgrowth, conductor, tegular apophysis, RTA (Fig. 19a–c) and lateral teeth (Fig. 20a and Fig. 21b), but can be distinguished by the embolus with a large seam from the middle to the tip (Fig. 19b, arrowed in Fig. 28c; absent in *B.yassassin*), by the median plate with distinct humped areas best seen in posterior view (Fig. 21b; absent in *B.yassassin*), by the spermathecae separated by less than their diameter, larger chamber round and apically swollen (Fig. 20b; spermathecae separated by about their diameter, larger chamber oval in *B.yassassin*) and by the fertilisation ducts pointing medially (Fig. 20b; fertilisation ducts pointing postero-medially in *B.yassassin*).

#### Etymology

The specific name refers to the type locality and is a noun in apposition.

#### Distribution

Vietnam (Vinh Phuc, type locality; Fig. 1).

### Bowie borneo

S. Li & Yao sp. n.

0ABF3BD1-8C4A-56D1-8838-1F91D4F3A751

4E110510-3EF4-4784-BE04-160B368F891B

#### Materials

- **Type status:** Holotype. **Occurrence:** recordedBy: Z. Chen; individualCount: 1; sex: male; lifeStage: adult; **Taxon:** order: Araneae; family: Ctenidae; genus: Bowie; **Location:** country: Malaysia; stateProvince: Borneo; verbatimLocality: State of Sabah, Mount Trus Madi, Jungle Girl Camp; verbatimElevation: 1234 m a.s.l.; verbatimLatitude: 5°33.000'N; verbatimLongitude: 116°30.960'E; **Event:** samplingProtocol: Collected by hand in leaf litter; year: 2016; month: 4; day: 27; **Record Level:** institutionCode: IZCAS-Ar 43730

#### Description

**Male** (IZCAS-Ar 43730): PL 3.8, PW 2.9, AW 1.2, OL 3.2, OW 2.2. Eye diameters and interdistances: AME 0.17, ALE 0.15, PME 0.22, PLE 0.21, AME–AME 0.14, AME–ALE 0.11, PME–PME 0.24, PME–PLE 0.27, AME–PME 0.11, ALE–PLE 0.13, clypeus AME 0.09, clypeus ALE 0.37. Palp and leg measurements: palp 5.1 (2.5, 0.5, 0.8, -, 1.3), I 12.1 (2.8, 1.5, 3.2, 3.3, 1.3), II 10.9 (3.2, 1.3, 2.6, 2.7, 1.1), III 9.6 (2.7, 1.1, 2.1, 2.6, 1.1), IV 15.1 (4.0, 1.4, 3.3, 4.7, 1.7). Leg formula 4123. Spination of palp and legs: palp 151, 100, 1110; femora I p012, d111, r012, II–III p112, d111, r112, IV p112, d111, r002; patellae I–II 100, III–IV 101; tibiae I p010, r010, v212222, II p100, d001, r110, v22222, III–IV p11, d111, r11, v222; metatarsi I p111, r011, v222, II p111, d002, r111, v222, III p111, d012, r111, v222, IV p111, d012, r111, v2212. Chelicerae with 3 promarginal, 4 retromarginal teeth and with elongated patch of 8 tiny denticles along entire cheliceral furrow. Retromargin of chelicerae close to fang base with 4 bristles. Sparse scopula on all tarsi. Leg claws I–II with 5 and III–IV with 4 secondary teeth. Position of tarsal organ: I 1.07, II 0.89, III 0.72, IV 1.06.

**Figure 22.**
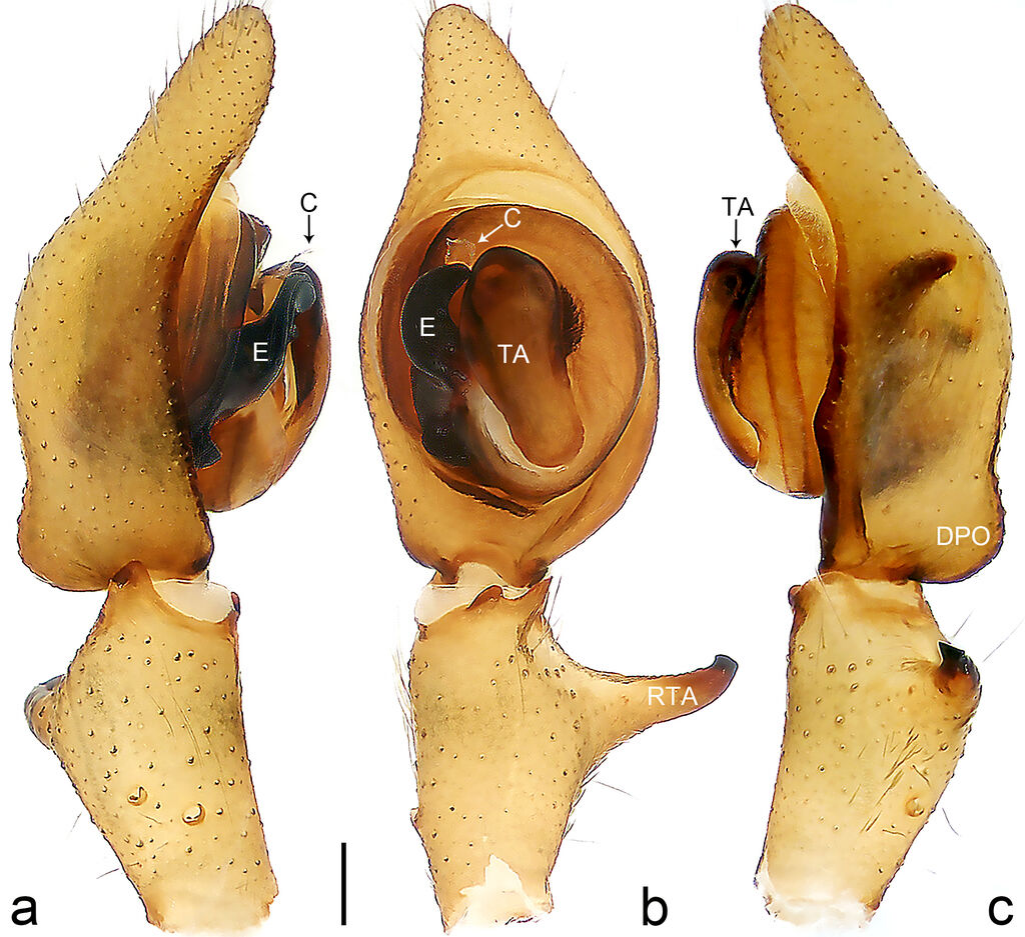
*Bowieborneo* sp. n., palp, holotype. **a**: Prolateral view; **b**: Ventral view; **c**: Retrolateral view. C = conductor, DPO = dorso-proximal outgrowth of cymbium, E = embolus, RTA = retrolateral tibial apophysis, TA = tegular apophysis. Scale bar: 0.2 mm (**a–c**).

Palp (Fig. 22a–c). RTA protruding at an almost right angle from tibia in ventral view, with slightly hooked tip. Cymbium tip slightly conical and with weakly developed dorso-proximal outgrowth. Embolus (Fig. 28d) arising at 7 o’clock position. Conductor arising at 11 o’clock position. Tegular apophysis arising from tegulum sub-proximally, with narrow base and wide tip.

**Figure 23.**
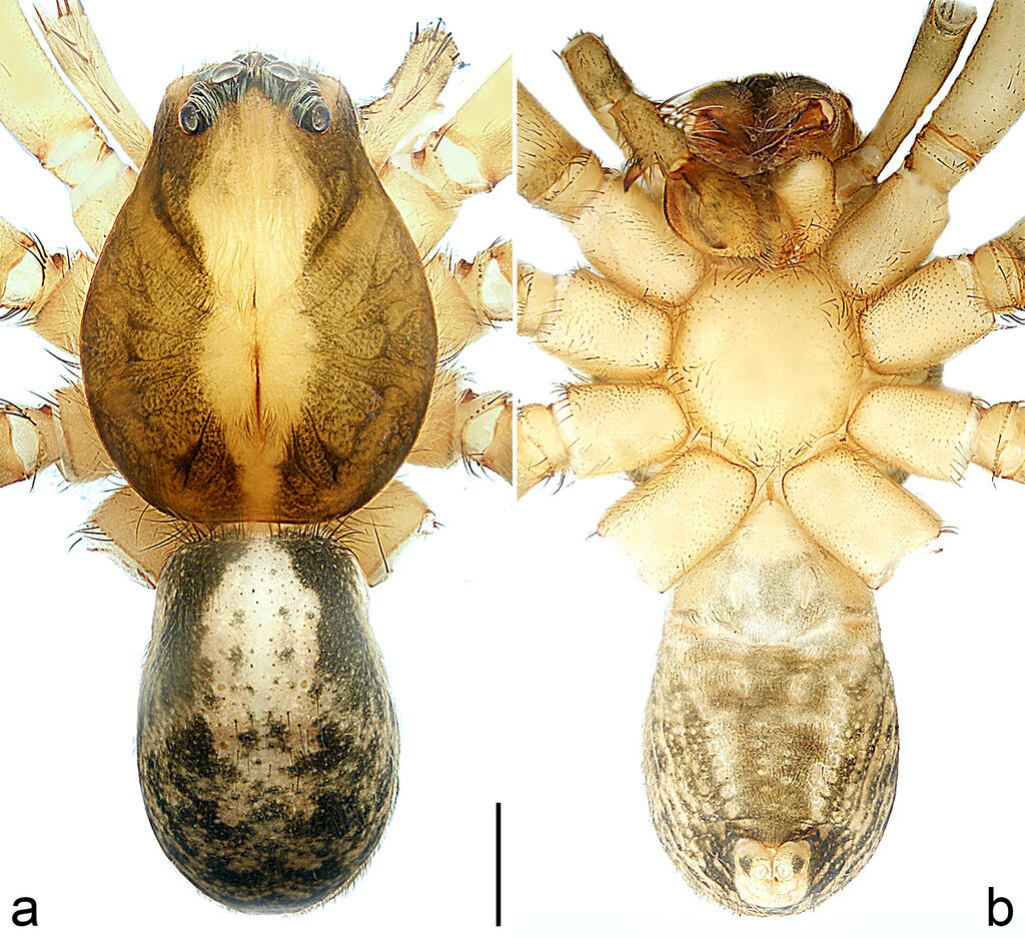
*Bowieborneo* sp. n., habitus, holotype male. **a**: Dorsal view; **b**: Ventral view. Scale bar: 1.0 mm (**a**, **b**).

Colour (Fig. 23a and b). Yellowish-brown with darker patterns. Dorsal prosoma with characteristic lighter median band, widened behind eyes, eye field with sparse white hairs, with distinctly marked fovea and distinct radial markings. Sternum, labium, gnathocoxae and ventral coxae yellowish-brown without patterns. Chelicerae reddish-brown with longitudinal patterns. Palps and legs yellowish-brown, legs II–IV with distinct patterns. Dorsal opisthosoma yellowish with black patches, anterior margin and central region light. Lateral opisthosoma spotted. Ventral opisthosoma yellowish-brown with posteriorly converging lines of spots. Anterior lateral spinnerets laterally dark, posterior lateral and median spinnerets and anal tubercle light.

**Female**

Unknown.

#### Diagnosis

Small Ctenidae (total length male 7.0). The new species is assigned to the *chinagirl*-species group with the characteristics of compact embolus, thick-walled, with rounded edges and with an entire margin, the tegular apophysis is longitudinally elongated, not covering the embolus. It resembles *B.abdulmajid* Jäger, 2022 (see Jäger 2022: figs 638–643 and 696–699) by having similar dorso-proximal cymbial outgrowth, embolus and RTA (Fig. 22a–c and Fig. 28d), but can be distinguished by the base of tegular apophysis having no bulge (Fig. 22b; the base of tegular apophysis bulging beyond tegulum margin in *B.abdulmajid*), by the tibia having no distinct broad longitudinal ridge ventrally (Fig. 22b; tibia with distinct broad longitudinal ridge ventrally in *B.abdulmajid*) and by the conductor nearly quadrilateral (Fig. 22b; conductor nearly oval in *B.abdulmajid*).

#### Etymology

The specific name refers to the type locality and is a noun in apposition.

#### Distribution

Malaysia (Borneo, type locality; Fig. 1).

#### DNA Barcode

Male (IZCAS-Ar 43730):

GGTTTGGTGCTTGGGCTTCTATAGCAGGTACGTCTATAAGAGTTTTGATTCGAATGGAATTAGGACATTCTGGAAGATTATTAGGGGATGATCATTTATATAATGTTGTTGTTACTGCTCATGCTTTTGTTATAATTTTTTTTATAGTGATACCTATTTTAATTGGTGGTTTTGGAAATTGGTTGGTTCCTTTAATATTAGGAGCTCCTGATATATCTTTTCCTCGTATAAATAATTTGTCGTTTTGATTACTTCCTCCTTCTTTATTTTTATTGTTTATATCTTCTATGACTGAGATAGGGGTGGGAGCTGGTTGGACGGTTTATCCACCTTTGGCTTCTGGAATTGGTCATGCAGGAAGATCGATAGATTTTGCTATCTTTTCTCTCCATTTAGCAGGTGCTTCTTCTATTATAGGAGCTATTAATTTTATTTCTACGATTATTAATATACGATTATTAGGAATGAGAATGGAAAAGGTTCCTTTGTTTGTATGGTCTGTTTTTATTACTGCAGTTTTATTATTATTATCTTTACCTGTTTTAGCGGGTGCTATTACTATATTATTAACGGATCGTAATTTTAATACTTCTTTTTTCGATCCTGCTGGAGGAGGGGATCCAATTTTATTTCAACATTTATTTTGATTTTTTG (GenBank accession number OP572103).

### Bowie engkilili

S. Li & Yao sp. n.

AF071857-D7A5-50D3-A3CA-F8671A065CE1

076F7CEC-CEB1-4DF4-92E2-B2DD56FFA36E

#### Materials

- **Type status:** Holotype. **Occurrence:** recordedBy: Z. Bai; individualCount: 1; sex: female; lifeStage: adult; **Taxon:** order: Araneae; family: Ctenidae; genus: Bowie; **Location:** country: Malaysia; locality: Engkilili; verbatimElevation: 5 m a.s.l.; verbatimLatitude: 1°6.367'N; verbatimLongitude: 111°43.933'E; **Event:** samplingProtocol: Collected by hand in leaf litter; year: 2018; month: 9; day: 10; **Record Level:** institutionCode: IZCAS-Ar 43731

#### Description

**Male**

Unknown.

**Female** (IZCAS-Ar 43731): PL 4.5, PW 3.6, AW 2.3, OL 3.9, OW 2.3. Eye diameters and interdistances: AME 0.21, ALE 0.11, PME 0.26, PLE 0.23, AME–AME 0.15, AME–ALE 0.31, PME–PME 0.27, PME–PLE 0.35, AME–PME 0.13, ALE–PLE 0.17, clypeus AME 0.14, clypeus ALE 0.41. Palp and leg measurements: palp 4.7 (1.6, 0.9, 1.1, -, 1.1), I missing, II 10.8 (3.0, 1.7, 2.7, 2.5, 0.9), III 10.2 (2.7, 1.7, 2.2, 2.5, 1.1), IV 14.4 (3.7, 1.6, 3.4, 4.2, 1.5). Leg formula 4123. Spination of palp and legs: palp 131, 001, 112, 2012; femora II p110, d111, r112, III p012, d111, r112, IV p002, d111, r112; patellae II 000, III–IV 101; tibiae II v22222, III–IV p11, d111, r11, v222; metatarsi II v222, III p112, d010, r112, v222, IV p112, d010, r112, v2222. Chelicerae with 3 promarginal, 4 retromarginal teeth and with elongated patch of 5 tiny denticles along entire cheliceral furrow. Retromargin of chelicerae close to fang base with 5 bristles. Sparse scopula restricted almost entirely to tarsi. Palpal claw with 7 secondary teeth, leg claws II–IV with 3 secondary teeth. Position of tarsal organ: II 0.75, IV 0.85.

**Figure 24.**
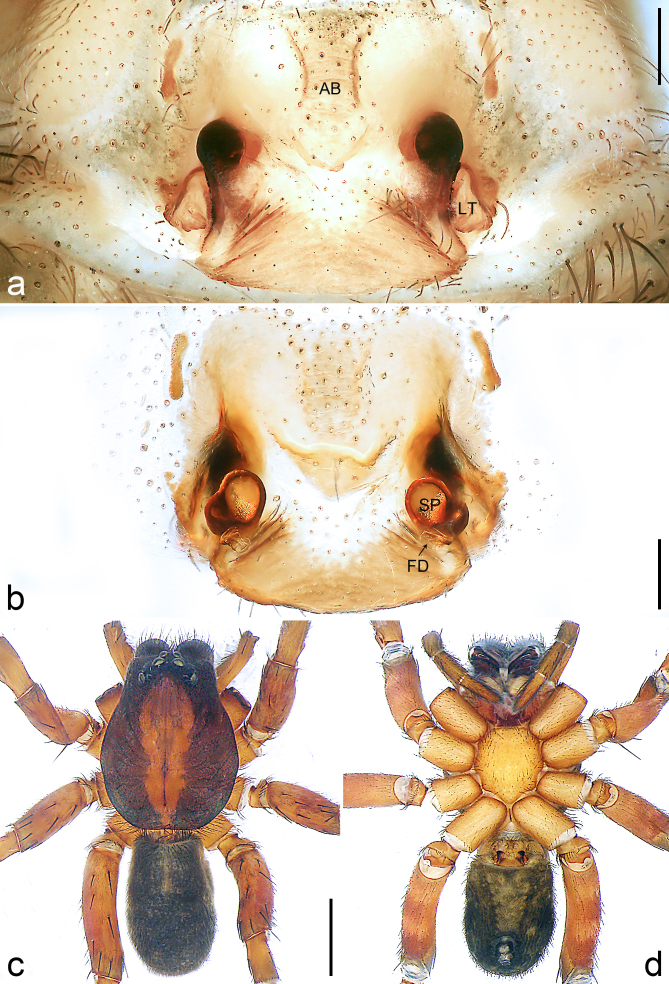
*Bowieengkilili* sp. n., holotype female. **a**: Epigyne, ventral view; **b**: Vulva, dorsal view; **c**: Habitus, dorsal view; **d**: Habitus, ventral view. AB = anterior bulge of median plate, FD = fertilisation duct, LT = lateral teeth, SP = spermathecae. Scale bars: 0.2 mm (**a**, **b**), 2.0 mm (**c**, **d**).

**Figure 25.**
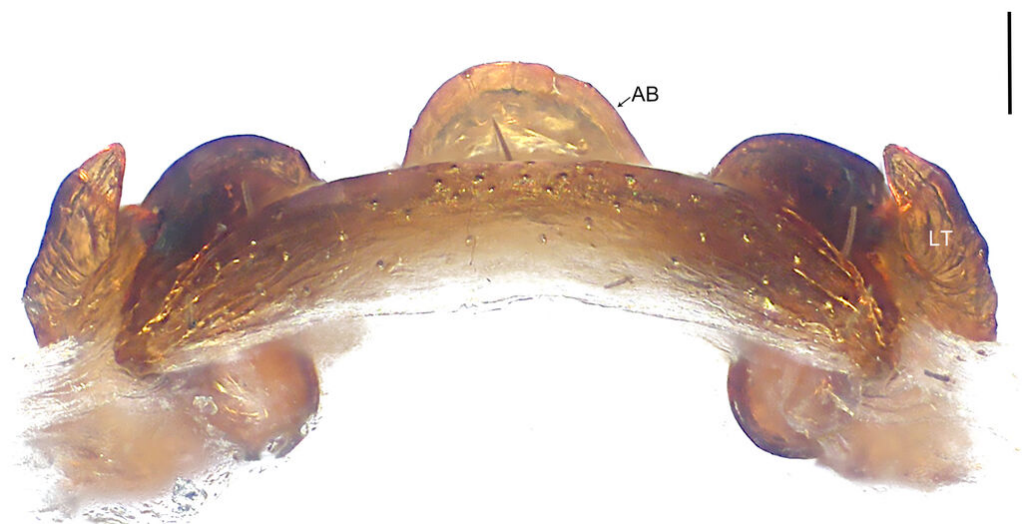
*Bowieengkilili* sp. n., epigyne, posterior view. AB = anterior bulge of median plate, LT = lateral teeth. Scale bar: 0.1 mm.

Copulatory organ (Fig. 24a, b and Fig. 25). Epigynal field slightly longer than wide, with narrow separated anterior bands laterally. Median plate with a median bulge in anterior half, laterally with two separate sclerotised patches, constricted at lateral teeth, the latter moderately pointed. Spermathecae small, separated by more than twice their diameter, roundish, smaller chamber bend at right angle, laterad.

Colour (Fig. 24c and d). Reddish-brown to yellowish with dark patterns. Dorsal prosoma with characteristic lighter median band, widened behind eyes, distinctly marked fovea and indistinct radial markings. Sternum and ventral coxae yellowish, labium and gnathocoxae reddish-brown. Chelicerae black. Leg reddish-brown. Dorsal and lateral opisthosoma black. Ventral opisthosoma black with light patterns. Spinnerets and anal tubercle black.

#### Diagnosis

Small Ctenidae (total length female 8.4). The new species resembles *B.withinyou* Jäger, 2022 (see Jäger 2022: figs 629–630 and 634–636) by having similar fertilisation ducts (Fig. 24b), but can be distinguished by the lateral teeth pointing medially (Fig. 24a; lateral teeth pointing postero-medially in *B.withinyou*), by the median plate with two separate sclerotised patches laterally and a longer median bulge in anterior half (Fig. 24a and Fig. 25; median plate with shorter median bulge in anterior half in *B.withinyou*) and by the spermathecae separated by more than twice their diameter (Fig. 24b; spermathecae separated by 1.5 times their diameter in *B.withinyou*).

#### Etymology

The specific name refers to the type locality and is a noun in apposition.

#### Distribution

Malaysia (Engkilili, type locality; Fig. 1).

#### DNA Barcode

Female (IZCAS-Ar 43731):

TATTTGGGGCTTGAGCTTCTATAGCTGGTACATCTATAAGTGTTTTGATTCGTATGGAGTTGGGACATTCGGGGAGAATATTGGGAGATGATCATCTCTATAATGTTATTGTTACTGCTCATGCTTTTGTTATAATTTTTTTTATAGTTATACCTATTTTAATTGGAGGTTTTGGTAATTGGTTGGTTCCTTTAATGTTAGGGGCTCCTGATATGTCGTTTCCTCGAATAAATAATTTGTCTTTTTGGTTACTTCCTCCTTCTTTATTTTTATTGTTTATGTCTTCTATAACTGAAATAGGGGTAGGAGCTGGTTGAACGGTGTATCCTCCTTTGGCTTCAAGAATGGGTCATGCTGGTAGATCTATGGATTTTGCTATTTTTTCTCTTCATTTAGCTGGGGCGTCTTCTATTATAGGTGCTATTAATTTTATTTCTACTATTATTAATATGCGTTTATTAGGAATGAGAATAGAGAAAGTTCCTTTGTTTGTATGGTCTGTTTTTATTACTGCGGTATTGTTATTATTGTCTCTTCCTGTTTTGGCAGGAGCTATTACTATATTGTTAACTGATCGTAATTTTAATACTTCTTTTTTTGATCCGGCTGGAGGGGGAGATCCGATTTTATTTCAACATTTATTTTGATTTTTT (GenBank accession number OP572106).

### Bowie sabah

S. Li & Yao sp. n.

0745AC4D-6E66-5AF6-ACB4-590F844F7BCB

D4FF31EC-AD04-4C90-A0EA-3AFF90C7B8A5

#### Materials

- **Type status:** Holotype. **Occurrence:** recordedBy: Z. Chen; individualCount: 1; sex: male; lifeStage: adult; **Taxon:** order: Araneae; family: Ctenidae; genus: Bowie; **Location:** country: Malaysia; stateProvince: Borneo; verbatimLocality: State of Sabah, Mount Trus Madi, Jungle Girl Camp; verbatimElevation: 1234 m a.s.l.; verbatimLatitude: 5°33.000'N; verbatimLongitude: 116°30.960'E; **Event:** samplingProtocol: Collected by hand in leaf litter; year: 2016; month: 5; day: 3; **Record Level:** institutionCode: IZCAS-Ar 43732
- **Type status:** Paratype. **Occurrence:** recordedBy: Z. Chen; individualCount: 1; sex: female; lifeStage: adult; **Taxon:** order: Araneae; family: Ctenidae; genus: Bowie; **Location:** country: Malaysia; stateProvince: Borneo; verbatimLocality: State of Sabah, Mount Trus Madi, Jungle Girl Camp; verbatimElevation: 1234 m a.s.l.; verbatimLatitude: 5°33.000'N; verbatimLongitude: 116°30.960'E; **Event:** samplingProtocol: Collected by hand in leaf litter; year: 2016; month: 5; day: 3; **Record Level:** institutionCode: IZCAS-Ar 43733

#### Description

**Male** (IZCAS-Ar 43732): PL 6.4, PW 5.1, AW 2.3, OL 4.8, OW 3.6. Eye diameters and interdistances: AME 0.22, ALE 0.17, PME 0.27, PLE 0.23, AME–AME 0.25, AME–ALE 0.40, PME–PME 0.34, PME–PLE 0.49, AME–PME 0.17, ALE–PLE 0.29, clypeus AME 0.11, clypeus ALE 0.52. Palp and leg measurements: palp 8.2 (3.0, 1.2, 1.6, -, 2.4), I 20.5 (5.6, 2.6, 5.4, 5.1, 1.8), II 17.4 (5.1, 2.5, 4.3, 4.2, 1.3), III 14.5 (4.3, 2.2, 3.2, 3.7, 1.1), IV 22.4 (6.1, 2.2, 5.4, 6.8, 1.9). Leg formula 4123. Spination of palp and legs: palp 161, 001, 111; femora I p021, d111, r1111, II p112, d111, r112, III p001, d222, r112, IV p012, d111, r112; patellae I–IV 101; tibiae I p110, d111, r11, v22222, II p110, d11, r11, v22222, III–IV p11, d111, r11, v222; metatarsi I–II p111, d012, r111, v222, III p111, d002, r111, v222, IV p111, d012, r111, v222. Chelicerae with 3 promarginal, 4 retromarginal teeth and with elongated patch of 23 tiny denticles along entire cheliceral furrow. Retromargin of chelicerae close to fang base with 6 bristles. Sparse scopula on all tarsi and metatarsi I–III. Leg claws I with 5, II with 4, III with 5 and IV with 6 secondary teeth. Position of tarsal organ: I with 1.64, II 1.15, III 0.96, IV 1.28.

**Figure 26.**
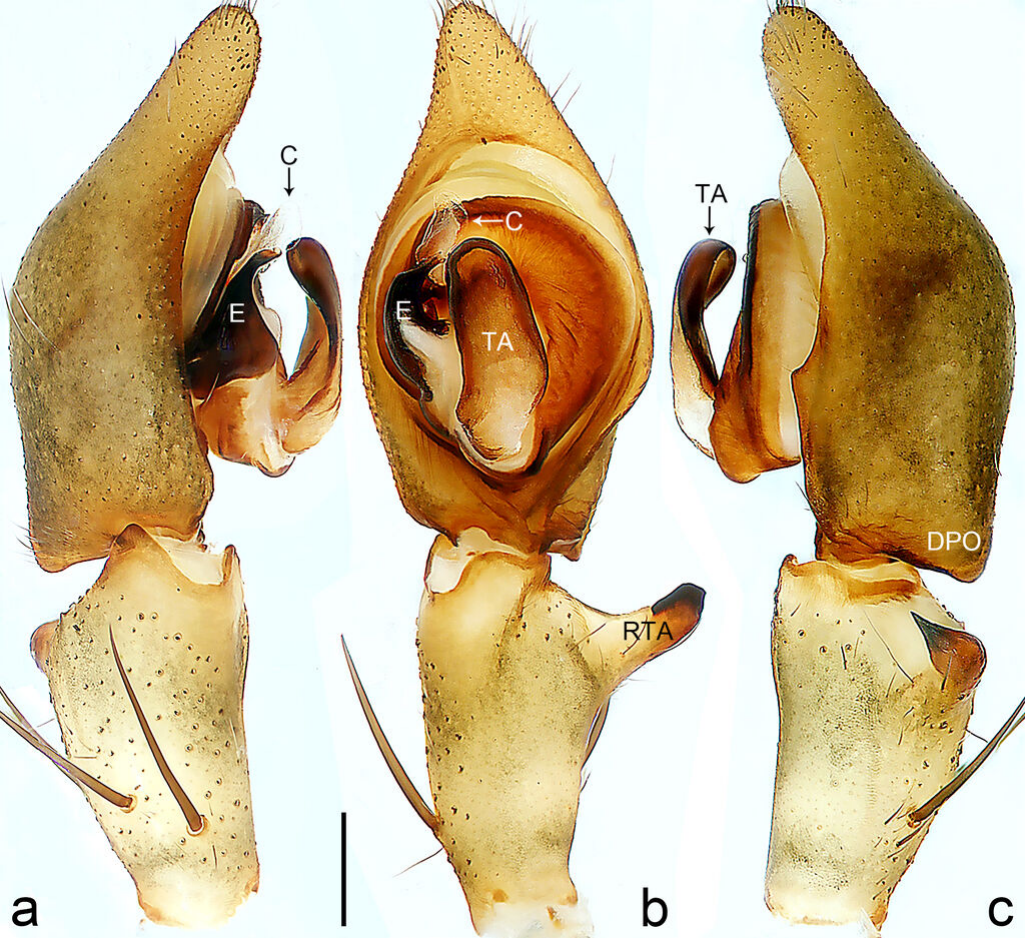
*Bowiesabah* sp. n., palp, holotype. **a**: Prolateral view; **b**: Ventral view; **c**: Retrolateral view. C = conductor, DPO = dorso-proximal outgrowth of cymbium, E = embolus, RTA = retrolateral tibial apophysis, TA = tegular apophysis. Scale bar: 0.5 mm (**a–c**).

Palp (Fig. 26a–c). RTA arising from tibia subdistally, with slightly hooked tip. Cymbium tip slightly conical and with weakly-developed dorso-proximal outgrowth. Embolus (Fig. 28e) arising at 8 o’clock position, with membranous extension at its base and with spermophor opening situated subapically. Conductor arising at 10 o’clock position. Tegular apophysis arising at 6 o’clock position, its base situated on a slightly proximally protruding part of tegulum.

**Figure 27.**
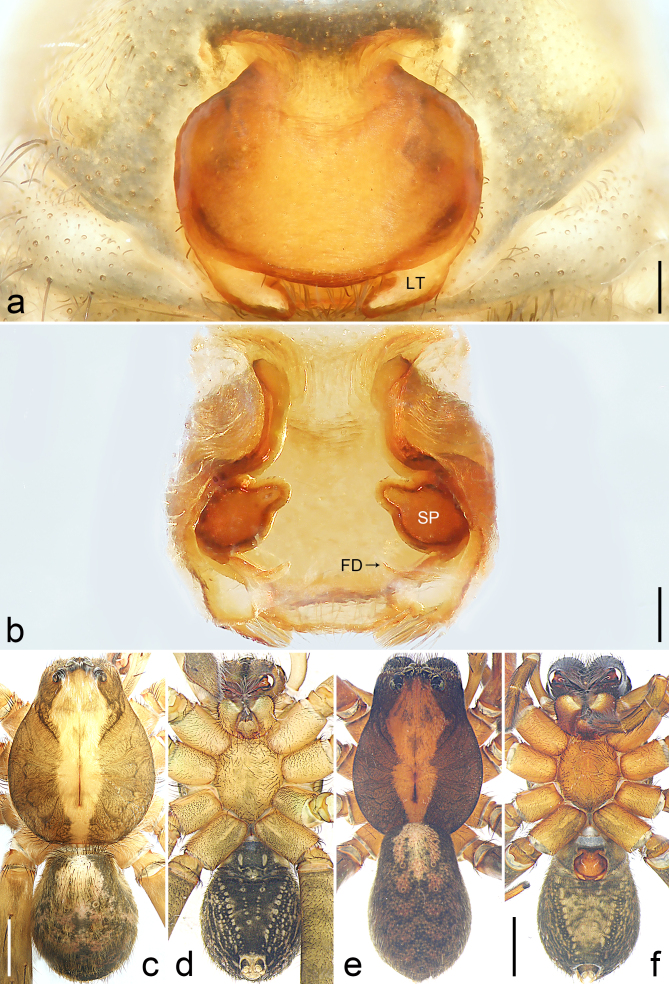
*Bowiesabah* sp. n. **a**: Paratype female, epigyne, ventral view; **b**: Same, vulva, dorsal view; **c**: Holotype male, habitus, dorsal view; **d**: Same, habitus, ventral view; **e**: Paratype female, habitus, dorsal view; **f**: Same, habitus, ventral view. FD = fertilisation duct, LT = lateral teeth, SP = spermathecae. Scale bars: 0.2 mm (**a**, **b**), 2.0 mm (**c–f**).

Colour (Fig. 27c and d). Yellowish-brown with darker patterns. Dorsal prosoma with characteristic lighter median band, widened behind eyes, eye field with sparse white hairs, with distinctly marked fovea and radial markings. Sternum and ventral coxae III + IV yellowish-brown with patches, coxae I + II yellowish-brown without patterns, labium and gnathocoxae yellowish-brown with lighter distal lips. Chelicerae dark yellowish-brown with longitudinal patterns. Palps and legs yellowish-brown, legs III + IV with distinct patterns. Dorsal opisthosoma yellowish-brown with black patches, anterior margin and central region light. Lateral opisthosoma spotted. Ventral opisthosoma dark brown with posteriorly converging lines of spots. Anterior lateral spinnerets laterally dark, posterior lateral and median spinnerets and anal tubercle light.

**Female** (IZCAS-Ar 43733): PL 5.9, PW 4.4, AW 3.0, OL 5.2, OW 3.5. Eye diameters and interdistances: AME 0.23, ALE 0.19, PME 0.27, PLE 0.25, AME–AME 0.20, AME–ALE 0.44, PME–PME 0.33, PME–PLE 0.52, AME–PME 0.17, ALE–PLE 0.28, clypeus AME 0.13, clypeus ALE 0.51. Palp and leg measurements: palp 5.8 (2.1, 1.1, 1.2, -, 1.4), I 13.9 (4.0, 2.1, 3.6, 3.1, 1.1), II 12.8 (3.8, 2.1, 3.0, 2.9, 1.0), III 11.3 (3.5, 1.7, 2.4, 2.7, 1.0), IV 16.7 (4.6, 1.9, 3.8, 4.9, 1.5). Leg formula 4123. Spination of palp and legs: palp 131, 001, 112, 203; femora I p021, d111, r011, II p012, d111, r111, III p112, d111, r112, IV p002, d111, r112; patellae I–II 000, III–IV 101; tibiae I–II v22222, III–IV p11, d111, r11, v222; metatarsi I–II v222, III–IV p111, d012, r111, v222. Chelicerae with 3 promarginal, 4 retromarginal teeth and with elongated patch of 12 tiny denticles along entire cheliceral furrow. Retromargin of chelicerae close to fang base with 7 bristles. Sparse scopula restricted almost entirely to tarsi, only metatarsi I–II with sparse scopula hairs. Palpal claw with 5 secondary teeth, leg claws I with 2, II with 3, III with 4 and IV with 3 secondary teeth. Position of tarsal organ: I 0.97, II 0.84, III 0.75, IV 1.06.

Copulatory organ (Fig. 27a and b). Epigynal field roughly as long as wide; constrictive anterior width/widest width: 0.53/1.15. Lateral teeth originating at posterior margin of median plate. Internal duct system with two large vulval folds laterally. Spermathecae bottle gourd-shaped. Vulval folds separated by less than the spermathecae length and subparallel medially. Fertilisation ducts pointing antero-medially.

Colour (Fig. 27e and f). As in male, except for being darker, reddish-brown. Chelicerae dark reddish-brown without pattern.

#### Diagnosis

Medium-sized Ctenidae (total length male 11.2, female 11.1). The new species is assigned to the *scarymonsters*-species group with the characteristics of embolus with a basal, ventral bulge (best seen in prolateral view), tegulum bulging proximally at tegular apophysis base, the subdistally arising, apically pointed RTA in males, transversally oval median plate and lateral teeth situated at posterior margin of epigyne and spermathecae bottle gourd-shaped. It resembles *B.neukoeln* Jäger, 2022 (see Jäger 2022: figs 440–446 and 448–460) by having similar dorso-proximal cymbial outgrowth, RTA (Fig. 26a–c) and lateral teeth (Fig. 27a), but can be distinguished by the embolus tip visible in ventral view (Fig. 26b and Fig. 28e; tegular apophysis covering the embolus tip in ventral view in *B.neukoeln*), by the tegular apophysis longitudinally orientated (Fig. 26b; tegular apophysis diagonally orientated in *B.neukoeln*), by the conductor nearly quadrilateral (Fig. 26b; conductor nearly elliptic in *B.neukoeln*) and by the constrictive anterior width/widest width: 1/2 (Fig. 27a; constrictive anterior width/widest width: 1/3 in *B.neukoeln*).

#### Etymology

The specific name refers to the type locality and is a noun in apposition.

#### Distribution

Malaysia (Borneo, type locality; Fig. 1).

#### DNA Barcode

Male (IZCAS-Ar 43732):

GGTTTGGAGCTTGAGCTTCTATAGTAGGAACATCTATAAGAGTATTAATTCGTATAGAATTAGGACATTCTGGAAGATTATTAGGAGATGATCATTTATATAATGTAGTTGTTACTGCTCATGCTTTTGTTATGATTTTTTTTATAGTAATGCCTATTTTAATTGGAGGTTTTGGAAATTGATTAGTTCCTTTAATATTAGGGGCTCCTGATATATCTTTTCCTCGAATAAATAATTTATCATTTTGATTACTTCCTCCTTCTTTATTCTTATTATTTATGTCTTCTATAACTGAGATAGGAGTAGGAGCTGGTTGAACAGTATATCCTCCCTTAGCTTCTAGAATAGGACATATGGGAAGATCAATGGATTTTGCTATTTTTTCTCTTCATTTAGCTGGGGCTTCCTCTATTATAGGAGCTATTAATTTTATTTCTACAATTATTAATATACGATTATTGGGAATAAGAATAGAGAAAGTTCCATTATTTGTGTGGTCTGTTTTTATTACTGCGGTATTGTTGTTATTGTCTTTACCTGTTTTAGCAGGTGCTATTACTATATTATTAACGGATCGTAATTTTAATACTTCTTTTTTTGACCCAGCTGGGGGGGGGGATCCCATTTTATTTCAACATTTATTTTGATTTTTGC (GenBank accession number OP572111).

Female (IZCAS-Ar 43733):

GGTTTGGAGCTTGAGCTTCTATAGTAGGAACATCTATAAGAGTATTAATTCGTATAGAATTAGGACATTCTGGAAGATTATTAGGAGATGATCATTTATATAATGTAGTTGTTACTGCTCATGCTTTTGTTATGATTTTTTTTATAGTAATGCCTATTTTAATTGGAGGTTTTGGAAATTGATTAGTTCCTCTAATATTAGGGGCTCCTGATATATCTTTTCCTCGAATAAATAATTTATCATTTTGATTACTTCCTCCTTCTTTATTCTTATTATTTATGTCTTCTATAACTGAGATAGGAGTAGGAGCTGGTTGAACAGTATATCCTCCCTTAGCTTCTAGAATAGGACATATGGGAAGATCAATGGATTTTGCTATTTTTTCTCTTCATTTAGCTGGGGCTTCCTCTATTATAGGAGCTATTAATTTTATTTCTACAATTATTAATATACGATTATTGGGAATAAGAATAGAGAAAGTTCCATTATTTGTGTGGTCTGTTTTTATTACTGCGGTATTGTTGTTATTGTCTTTACCTGTTTTAGCAGGTGCTATTACTATATTATTAACGGATCGTAATTTTAATACTTCTTTTTTTGACCCAGCTGGGGGGGGGGATCCCATTTTATTTCAACATTTATTTTGATTTTTGC (GenBank accession number OP572109).

#### Note

*B.sabah* sp. n. belongs to the *scarymonsters*-species group because of the abovementioned characteristics, so the “diagonally orientated TA” in the diagnosis of the *scarymonsters*-species group in Jäger (2022) should be changed to “tegulum bulging proximally at TA base”.

## Discussion

The posterior median eyes of all *Amauropelma* spiders from Asia are obviously larger than anterior median eyes, except for cave-dwelling species and *Am.yunnan* Yao & Li, 2022 (Jäger 2012, Miller and Rahmadi 2012, Chu et al. 2022, Lin et al. 2022). For the cave-dwelling species, for example, *Am.guangxi* Lin & Li, 2022 (Longgong Cave, China), *Am.krabi* sp. n. (Klang Cave, Thailand), *Am.matakecil* Miller & Rahmadi, 2012 (Anjani Cave, Indonesia), *Am.phangnga* sp. n. (Tapan Cave, Thailand) and *Am.saraburi* sp. n. (Tham Bo Pla Cave, Thailand), the PME and AME are almost the same size (Miller and Rahmadi 2012, Lin et al. 2022). These reduced PME may be the morphological adaptation of cave-dwelling spiders to caves. In particular, *Am.ekeftys* Jäger, 2012 (West Khasi Hills, India) also is a cave-dwelling species and its eyes are strongly reduced in size and pigments are absent (Jäger 2012). It is worth mentioning that the PME and AME of *Am.yunnan* Yao & Li, 2022 (litter layer, Xishuangbanna, China) are almost the same size (Chu et al. 2022), so these morphological characters need to be further studied in the future.

## Supplementary Material

XML Treatment for Amauropelma krabi

XML Treatment for Amauropelma phangnga

XML Treatment for Amauropelma saraburi

XML Treatment for Anahita medog

XML Treatment for Anahita popa

XML Treatment for Bowie fascination

XML Treatment for Bowie ninhbinh

XML Treatment for Bowie vinhphuc

XML Treatment for Bowie borneo

XML Treatment for Bowie engkilili

XML Treatment for Bowie sabah

## Figures and Tables

**Figure 9a.**
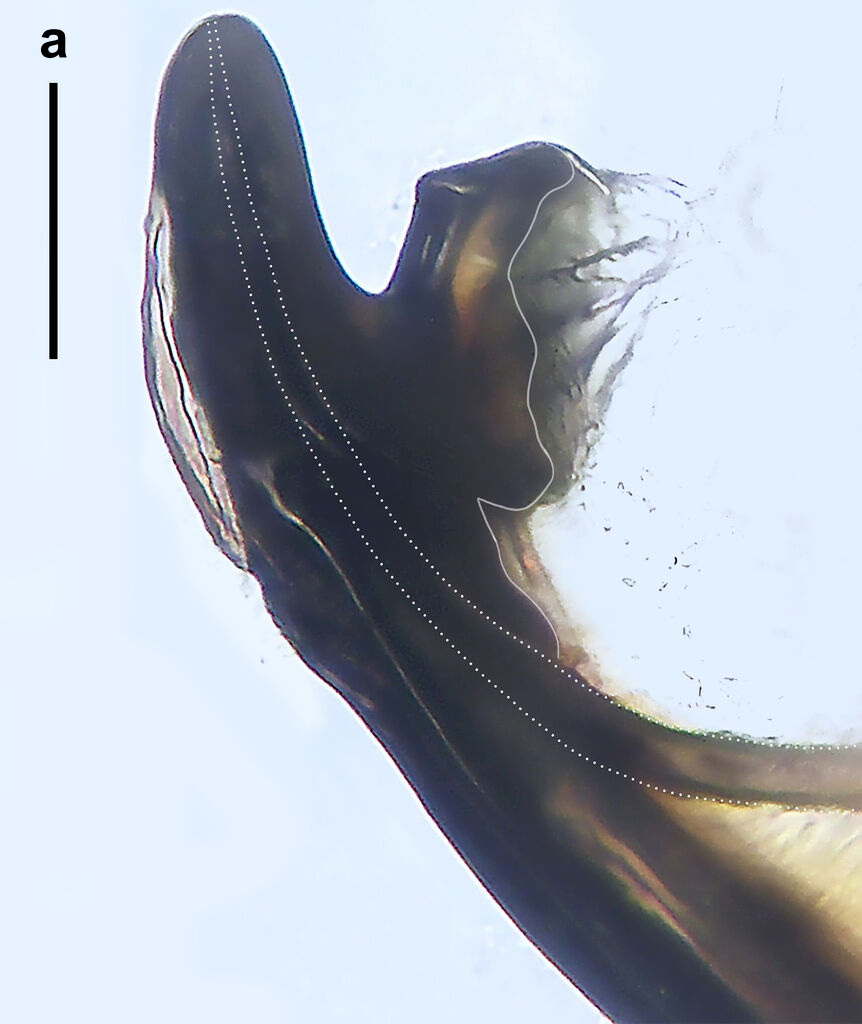
*Am.phangnga* sp. n., paratype. Scale bar: 0.05 mm.

**Figure 9b.**
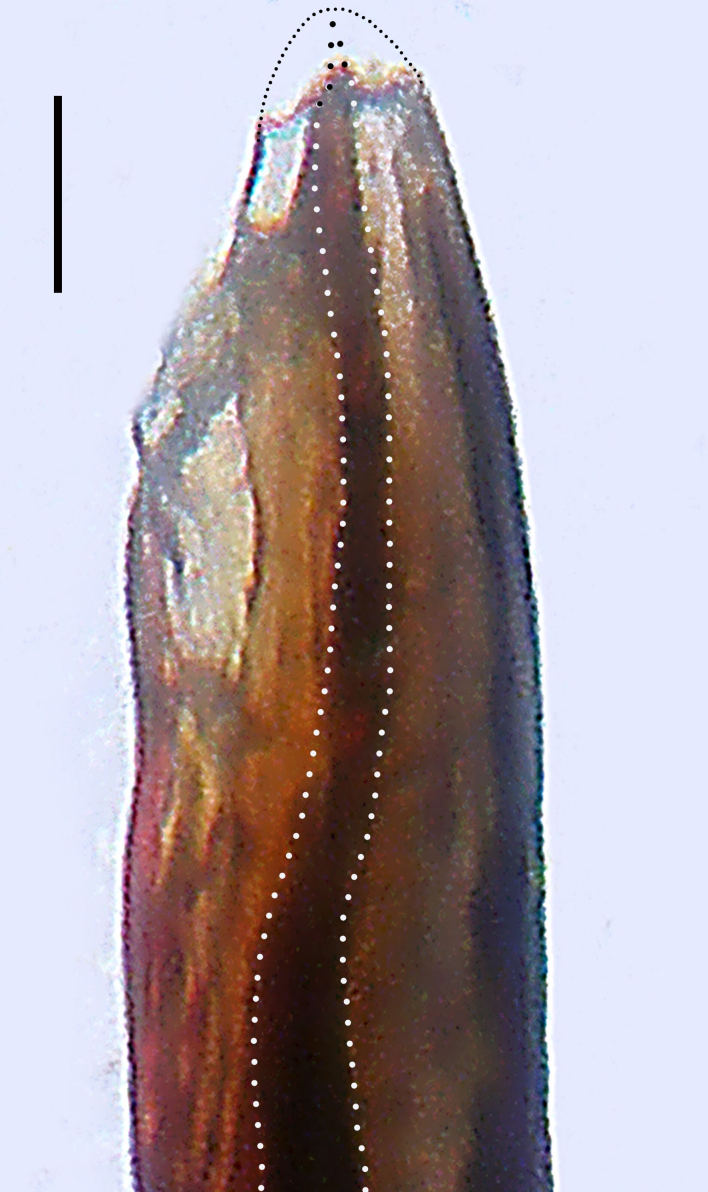
*Am.saraburi* sp. n., holotype. Scale bar: 0.02 mm.

**Figure 14a.**
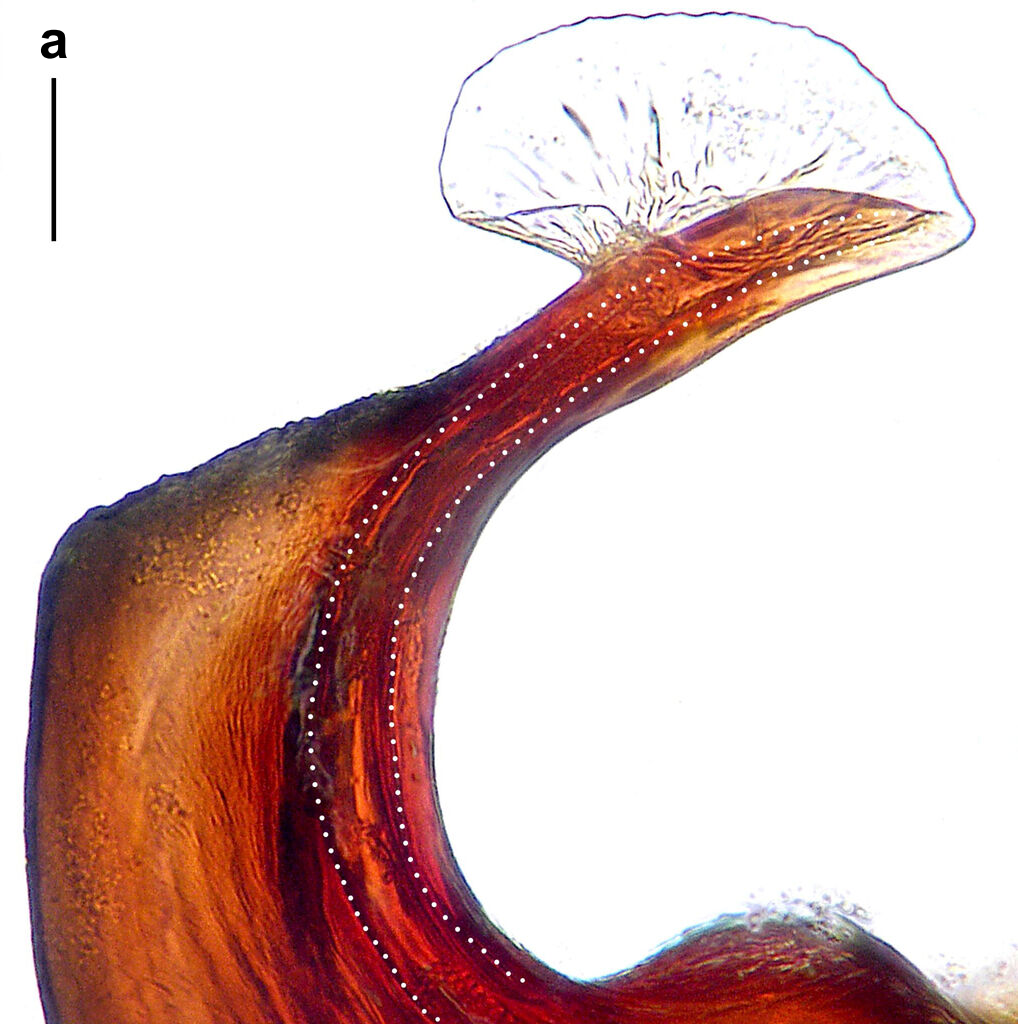
*An.medog* sp. n., holotype. Scale bar: 0.05 mm.

**Figure 14b.**
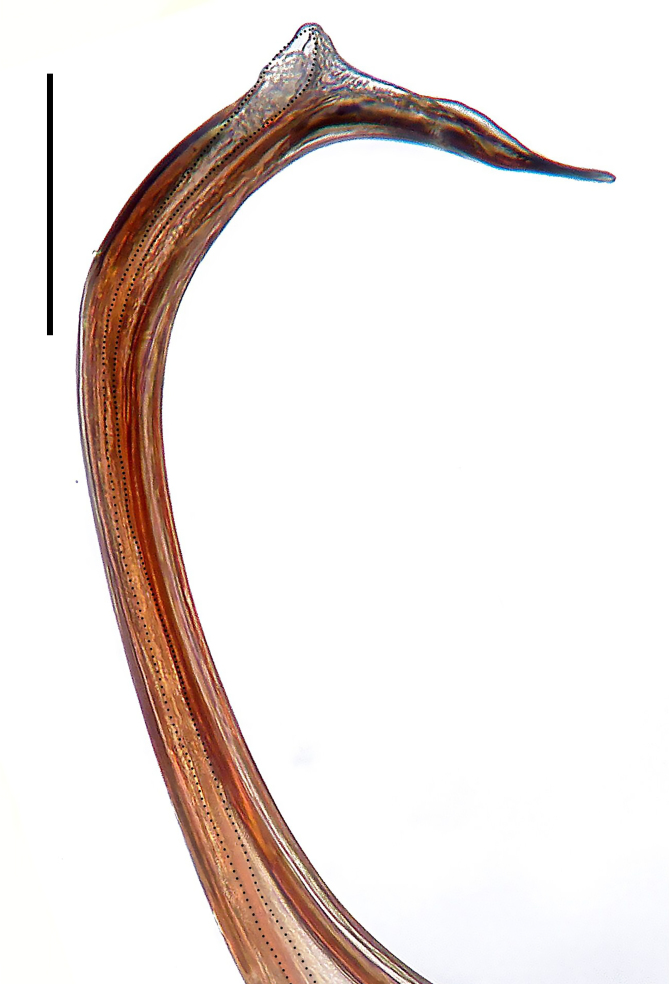
*An.popa* Jäger & Minn, 2015, male from Sagaing Hill. Scale bar: 0.01 mm.

**Figure 15.**
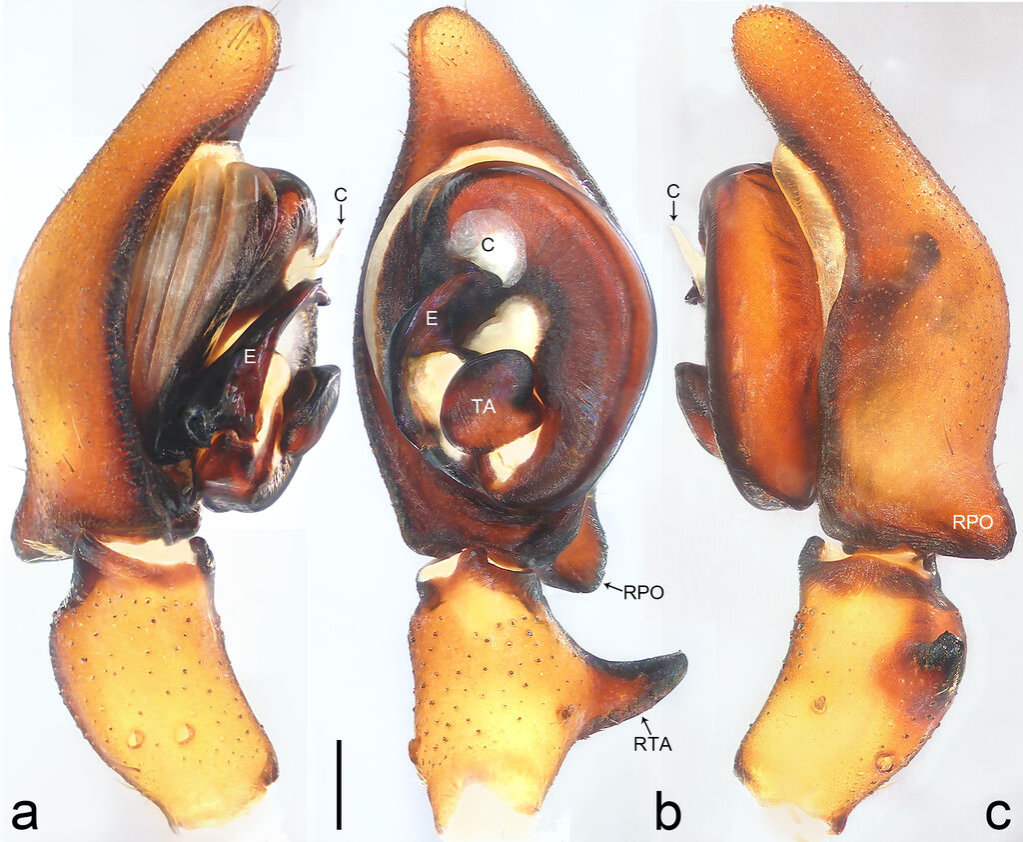
*Bowiefascination* Jäger, 2022, palp, male from Yunnan. **a**: Prolateral view; **b**: Ventral view; **c**: Retrolateral view. C = conductor, E = embolus, RPO = retro-proximal cymbial outgrowth, RTA = retrolateral tibial apophysis, TA = tegular apophysis. Scale bar: 0.5 mm (**a–c**).

**Figure 28a.**
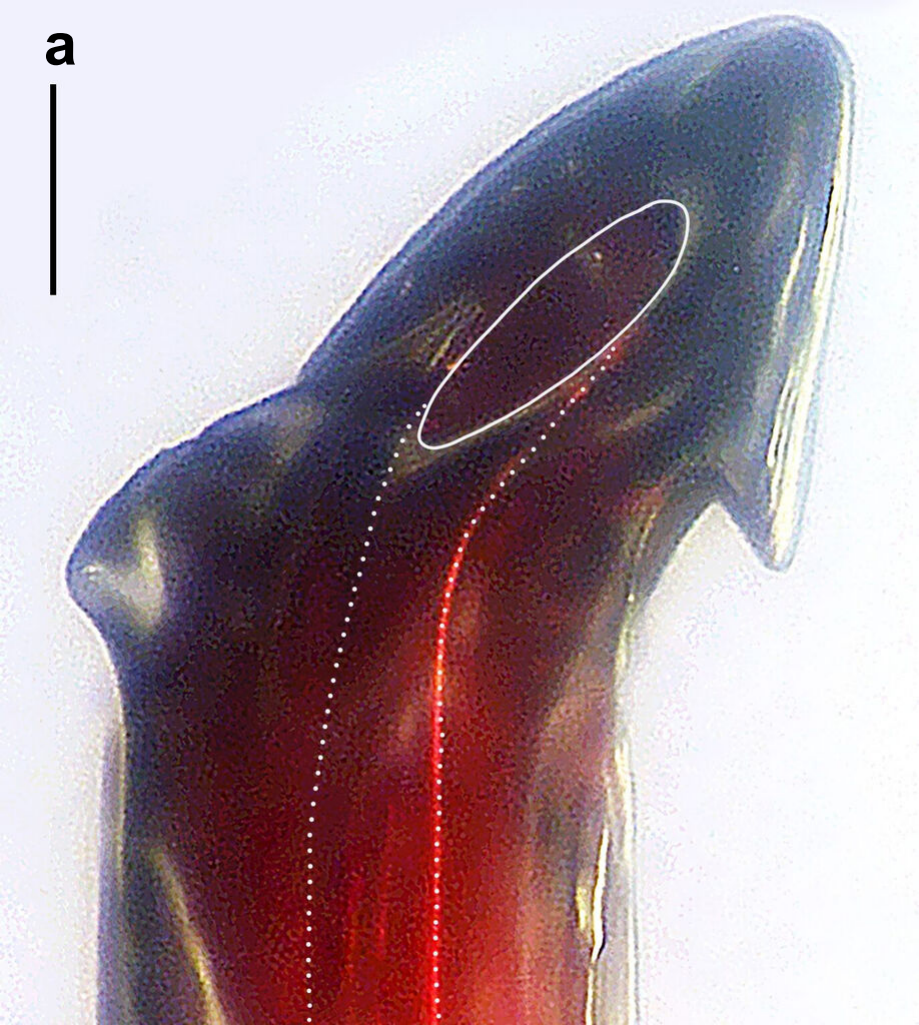
*B.fascination* Jäger, 2022, male from Yunnan, ventral view. Scale bar: 0.05 mm.

**Figure 28b.**
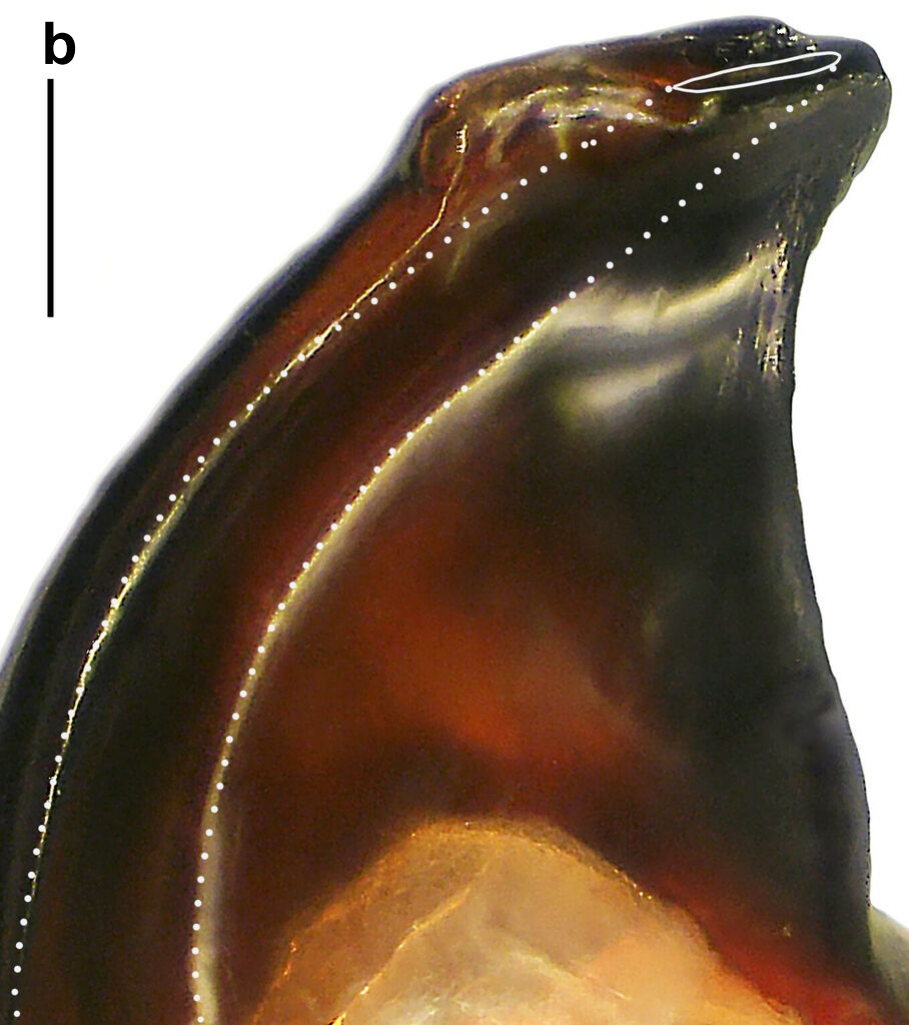
*B.ninhbinh* sp. n., paratype, ventral view. Scale bar: 0.1 mm.

**Figure 28c.**
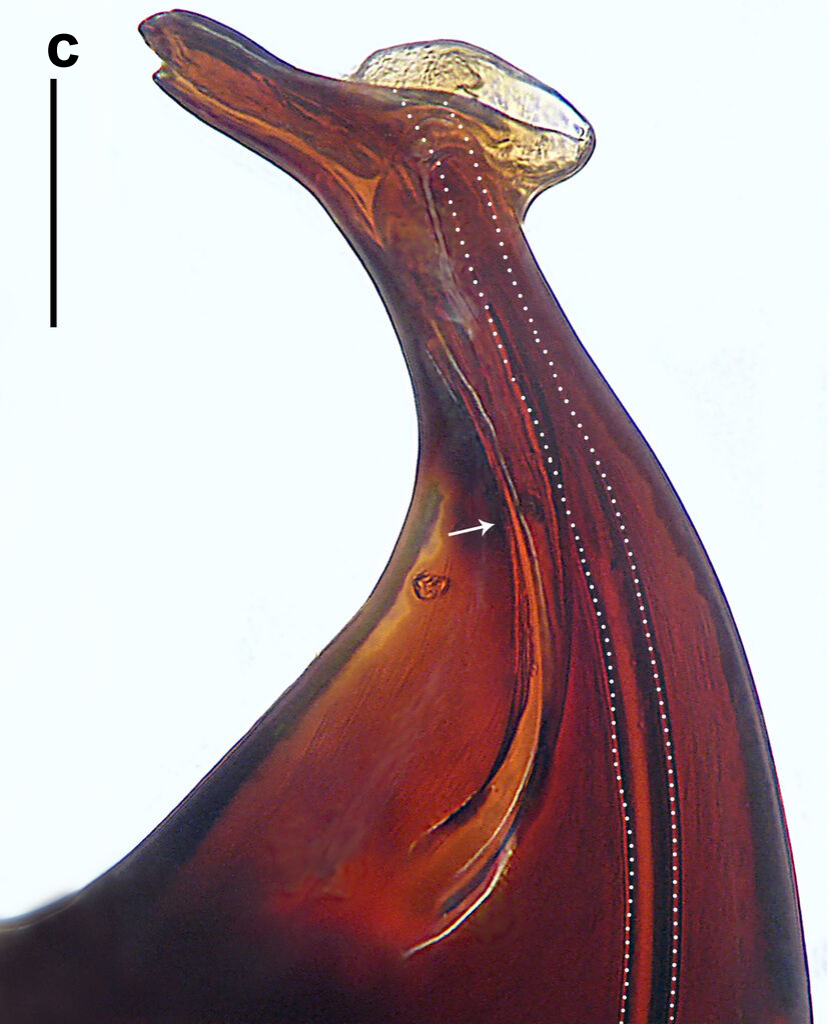
*B.vinhphuc* sp. n., paratype, dorsal view, arrow points at seam. Scale bar: 0.1 mm.

**Figure 28d.**
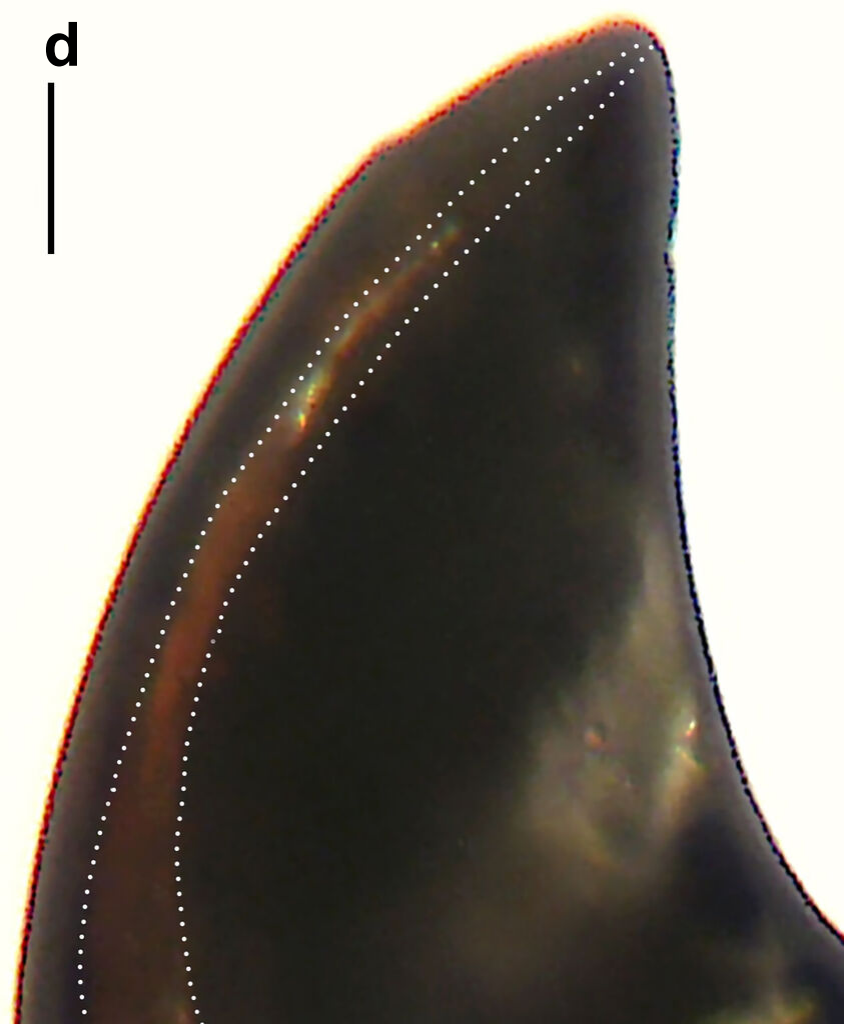
*B.borneo* sp. n., holotype, ventral view. Scale bar: 0.02 mm.

**Figure 28e.**
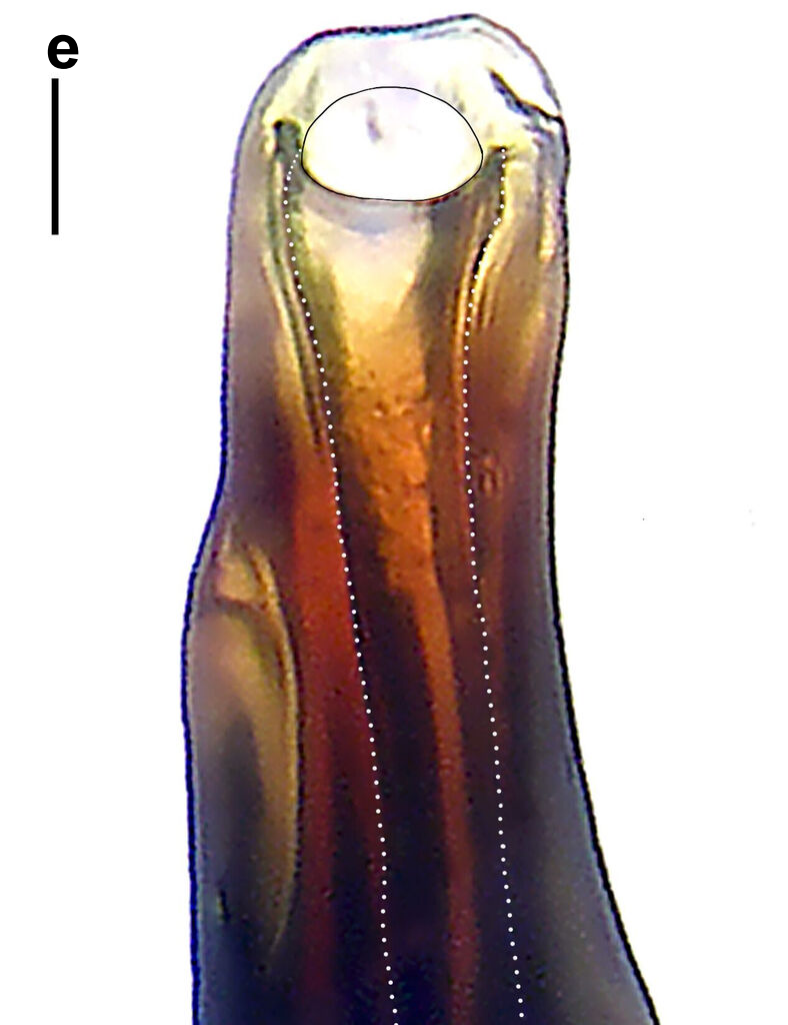
*B.sabah* sp. n., holotype, ventral view. Scale bar: 0.02 mm.
